# Transition Metal Compounds for Aqueous Ammonium‐Ion Batteries: Storage Mechanisms and Electrode Design

**DOI:** 10.1002/advs.202524166

**Published:** 2026-01-22

**Authors:** Can Li, Ziyuan Lan, Hanghang Liu, Yunxuan Jiang, Lingfeng Zhu, Xiaoning Li, Bo‐Tian Liu

**Affiliations:** ^1^ Guangxi Key Laboratory of Electrochemical and Magneto‐Chemical Functional Materials Guilin University of Technology Guilin China; ^2^ Centre For Atomaterials and Nanomanufacturing (CAN) School of Science RMIT University Melbourne VIC Australia

**Keywords:** ammonium‐ions batteries, anode material, aqueous batteries, cathode material, transition metal compounds

## Abstract

Aqueous ammonium‐ion batteries (AAIBs) have recently emerged as promising candidates for next‐generation energy storage owing to their intrinsic safety, environmental benignity, and cost efficiency. The unique tetrahedral configuration and hydrogen‐bonding capability of NH_4_^+^ enable fast ion transport and dendrite‐free operation, distinguishing AAIBs from traditional metal‐ion systems. However, sluggish NH_4_^+^ intercalation kinetics and electrode structure degradation have limited their practical implementation. Transition metal compounds (TMCs), with flexible oxidation states, rich redox activity, and tunable electronic structures, provide a versatile platform to address these issues. This review systematically summarizes recent progress in TMCs‐based electrodes for AAIBs, encompassing oxides, sulfides, carbides, nitrides, and other related compounds. We begin by distinguishing the operational principles of AAIBs in conventional “rocking‐chair” and dual‐ion configurations, emphasizing their distinct charge‐storage pathways and associated performance limitations. Subsequently, we elucidate the fundamental mechanisms governing ammonium‐ion storage and hydrogen‐bonding dynamics in governing ion transport. Finally, we outline a roadmap aimed at guiding future research efforts, offering material design insights into the commercialization of next‐generation safe and sustainable aqueous energy storage technologies. Unlike previous reviews that primarily focused on hydrogen bonding, organic electrodes, or safety chemistry, this review offers a material perspective that bridges inorganic redox chemistry with NH_4_^+^‐ion dynamics.

## Graphical abstract

Aqueous ammonium‐ion batteries leverage hydrogen‐bond‐mediated NH_4_^+^ storage in tunable transition metal compounds. Despite progress in Mn‐, V‐, Mo‐, and W‐based compounds, 2D LDHs, and MXenes, challenges like structural instability and slow kinetics persist. Future advances require robust host design, mechanistic understanding via operando studies, and innovative full‐cell architectures for practical, high‐performance AAIBs.

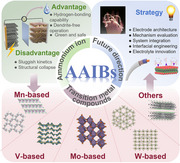

## 1 Introduction

**FIGURE 1:**
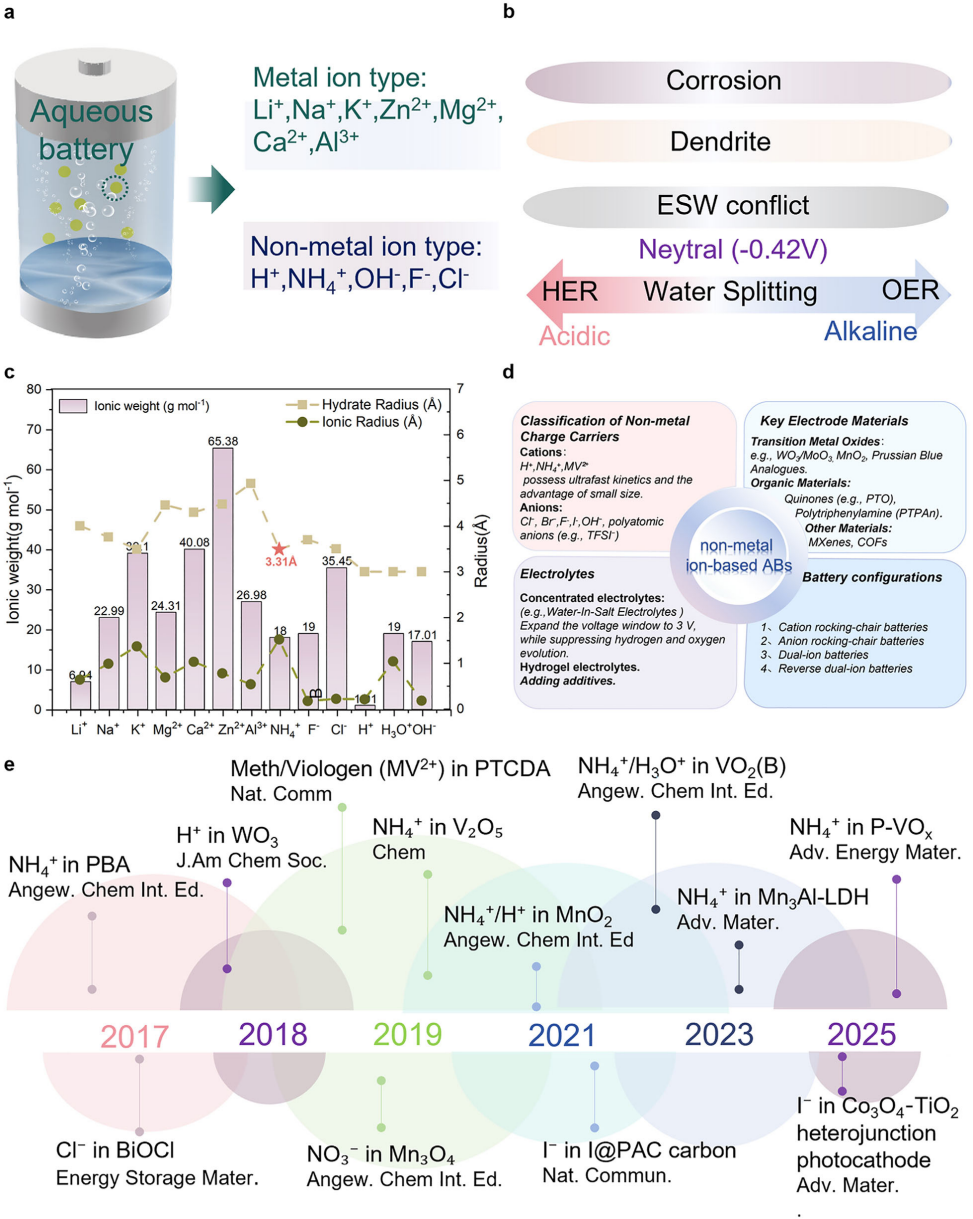
(a) Classification of ABs based on primary charge carriers, highlighting the distinction between metal‐ion and non‐metal‐ion systems. (b) The drawbacks of aqueous batteries using metal ions as charge carriers. (c) Comparative analysis of ionic weight, cation radius, and hydrated radius for typical metal‐ion (e.g., Li^+^, Na^+^) and non‐metal ion (e.g., OH^−^, NH_4_^+^, F^−^, H_3_O^+^) charge carriers, demonstrating the advantages of non‐metal ions in terms of lower molar mass and smaller hydrated radii, which facilitate rapid diffusion within aqueous electrolytes. (d, e) Summary of recent advances in non‐metal ion‐based ABs, showcasing key materials and their performance metrics [15, 16, 17, 18, 19, 20, 21] (Copyright 2017, Elsevier; 2018, ACS; 2019, Spring Nature; 2019, GDCh; 2021, Spring Nature; 2025, Wiley‐VCH).

Addressing global environmental challenges and mitigating the greenhouse effect necessitate the urgent development of environmentally friendly and sustainable energy sources. Current research efforts are focused on harnessing intermittent and stochastic renewable energy forms, including wind, solar, and tidal power [1, 2, 3, 4]. These renewable sources demand high‐efficiency energy storage solutions for peak shaving and valley filling to stabilize power supply fluctuations. Consequently, the increasing demand for electrochemical energy storage (EES) has propelled the advancement of diverse battery systems. Among these, rechargeable aqueous batteries (ABs) have emerged as particularly promising candidates for large‐scale energy storage applications due to their superior safety, cost‐effectiveness, and eco‐friendliness [5]. For example, ABs employing diverse charge carriers, such as utilizing charge carriers such as Li^+^ [6, 7], Na+ [7], K+ [8], Ca2+ [9], Mg^2+^ [8], Zn^2+^ [10, 11], and Al^3+^ [12], have been developed in recent years. These systems eliminate the safety risks associated with organic electrolytes while reducing reliance on critical metals through a multi‐ion strategy, thereby offering a more sustainable and cost‐effective route to grid‐scale energy storage. Nevertheless, the use of metallic ions as charge carriers in these ABs raises significant concerns, including corrosion, passivation, and dendrite formation, posing a challenge to sustainable development principles (Figure 1a,b) [13, 14].

This challenge has motivated the development of non‐metal ions‐based batteries, which are supposed to offer smaller hydrated ionic radii and lower molar masses, enabling faster diffusion kinetics in aqueous media (Figure 1a,c). Crucially, non‐metal ions are inherently resistant to dendrite formation, markedly improving safety and long‐term stability. Recent breakthroughs and key materials in non‐metal ion‐based ABs are summarized in Figure 1d,e [15, 16, 17, 18, 19, 20, 21]. Among these non‐metallic charge carriers, NH_4_^+^ is particularly noteworthy due to its lower corrosivity and reduced tendency toward hydrogen evolution reactions (HER), resulting in superior cycling performance compared to protons, halides, and hydronium ions. Additionally, the tetrahedral geometry and relatively moderate acidity of ammonium ions indicate that their conduction mechanism within electrode material lattices may significantly differ from that of traditional spherical metallic charge carriers. Besides, the structural features of ammonium ions imply that their insertion and de‐insertion in electrode materials likely involve more complex chemical bonding changes, such as the formation and cleavage of covalent‐ionic bonds, alongside the creation and disruption of hydrogen bonds [22, 23, 24]. These unique properties position aqueous ammonium‐ion batteries (AAIBs) as highly advantageous for achieving both high efficiency and stability while addressing the environmental and resource limitations commonly associated with metallic ion‐based systems. Consequently, AAIBs are particularly well‐suited for low‐cost energy storage systems designed for large‐scale smart grid applications [25, 26, 27].

Recent studies have investigated AAIBs from diverse perspectives, including safety chemistry, electrolyte design, hydrogen‐bond‐assisted transport, and organic electrode frameworks, as summarized in previous reviews [13, 22, 27, 28, 29, 30, 31]. Notably, Liu et al. provided a focused overview of cathode materials for ammonium‐ion batteries, with an emphasis on performance metrics and material screening [13]. In contrast, the present review goes beyond material‐level benchmarking to systematically analyze ammonium storage mechanisms across different families of transition metal compounds (TMCs), thereby offering a complementary and mechanistically driven perspective. While these efforts have deepened the understanding of NH_4_^+^‐related interfacial behavior and structural evolution, they predominantly center on molecular‐level or safety‐oriented aspects, leaving the fundamental interactions between the NH_4_^+^ and its corresponding electrode materials poorly understood. As high‐performance electrode candidates, TMCs exhibit rich redox chemistry, structural tunability, and flexible oxidation states that can accommodate NH_4_^+^ insertion and extraction. The interplay between crystal field environments, hydrogen bonding, and electronic configurations enables TMCs to provide both high reversibility and fast charge transfer [32]. However, a systematic understanding of how NH_4_^+^ interacts with TMC hosts, especially regarding charge storage mechanisms, lattice dynamics, and structure–property relationships, remains limited, and practical implementation is further challenged by sluggish intercalation kinetics, framework distortion, and interfacial instability.

In this context, this review aims to fill this gap by providing a comprehensive electrode material‐centred perspective on aqueous NH_4_^+^‐based storage devices. The charge storage mechanisms and battery configurations employed in AAIBs are first elucidated to highlight the unique physicochemical interactions between NH_4_^+^ and electrode hosts. Subsequently, recent advances in TMCs‐based electrodes, encompassing manganese‐, vanadium‐, molybdenum‐ and tungsten‐based compounds, two‐dimensional (2D) transition‐metal carbides/nitrides (MXenes), and layered double hydroxides (LDHs), are summarized. Key electrochemical metrics, including capacity, rate capability, cycling stability, operational voltage window, and cost‐effectiveness, are critically assessed for these electrodes. Finally, outlooks and design guidelines for constructing next‐generation AAIBs that bridge inorganic redox chemistry and NH_4_^+^‐ion dynamics are provided, offering insights into the rational design of safe, sustainable, and high‐performance aqueous energy storage systems.

## 2 Battery Configurations and Working Mechanisms

**FIGURE 2:**
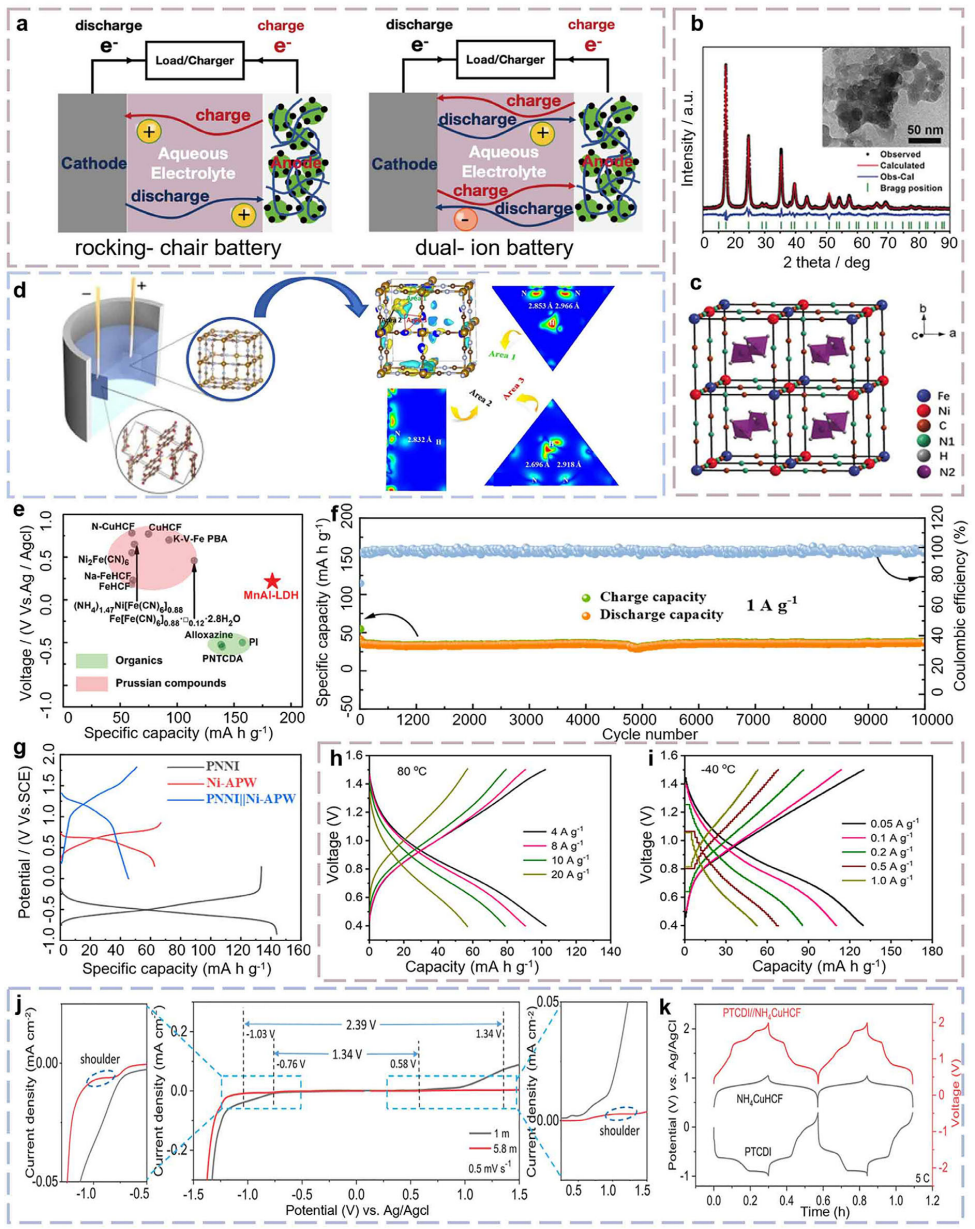
(a) Representative AAIBs categorized by storage mechanism, including rocking‐chair and dual‐ion storage mechanisms. (b) XRD pattern of Ni‐APW. (c) Perfectly arranged crystal lattice unit cell of Ni‐APW ^[^ ^34^ ^]^ (Copyright 2017, Wiley‐VCH). (d) 3D electron density difference (ρ‐ρ _corr._) and its 2D slices across, reveal hydrogen bonds formed between NH_4_^+^ and N species of PBA [35] (Copyright 2021, Elsevier). (e) Comparison of the discharge voltage plateau and specific capacity between Mn_3_Al‐LDH nanosheets and previously reported cathodes for aqueous ammonium battery [37] (Copyright 2023, Wiley‐VCH). (f) Cycling performance of PNNI//Ni‐APW at 1 A g–^1^ in 1 m NH_4_Cl electrolyte. (g) Rate performance of PNNI//Ni‐APW [38] (Copyright 2023, ACS). (h, i) Electrochemical performance of the NiHCF@CNTs//poly(1,5‐naphthalenediamine) full cell at −40°C and 80°C, respectively, under various current densities [39] (Copyright 2021, Elsevier). (j) Electrochemical stability window of the diluted 1 m (NH_4_)_2_SO_4_ electrolytes (LCE) and diluted 5.8 m (NH_4_)_2_SO_4_ electrolytes (HCE) determined by linear sweep voltammetry (LSV). (k) GCD profiles during the initial two cycles of Cu‐based PBA (N‐CuHCF) half‐cell, PTCDI half‐cell, and PTCDI//N‐CuHCF full cell using high‐concentration electrolyte at 300 mA g^−1^ [40] (Copyright 2022, Wiley‐VCH).

Understanding fundamental battery configurations is essential for optimizing electrode material design and interfacial kinetics in AAIBs. Based on the migration behavior and type of charge carriers within the electrolyte, AAIBs can be broadly classified into two distinct operational configurations: (1) rocking‐chair batteries and (2) dual‐ion batteries (DIBs), as illustrated in Figure 2a and summarized in Table 1. In rocking‐chair batteries, the NH_4_^+^ ions serve as the sole mobile charge carriers, reversibly shuttling between the cathode and anode during charge/discharge cycles. This configuration features well‐defined ion diffusion pathways and relatively stable electrode–electrolyte interfaces, which collectively contribute to high Coulombic efficiency and excellent long‐term cycling stability. In contrast, DIBs operate through the simultaneous migration of cations and anions from the electrolyte: NH_4_^+^ cations move toward the anode while anions (e.g., SO_4_^2−^ or NO_3_^−^) migrate toward the cathode. This decoupled ion transport enables higher cell voltages and expands the range of compatible electrode materials. However, it also introduces challenges such as complex interfacial side reactions, sluggish ion diffusivity, and reduced structural reversibility in host materials. Clarifying these two mechanistic paradigms provides a critical framework for evaluating material performance, guiding electrode design, and developing next‐generation AAIBs.

**TABLE 1:** Comparison of cathode/anode materials, electrolyte compatibility, and performance in rocking‐chair and dual‐ion AAIBs.

	Host material	Electrolyte	Working voltage	Capacity	Energy/power density	Capacity retention	Ref
Rocking‐chair batter	Ni‐APW//PTCDI	1 m aqueous (NH_4_)_2_SO_4_	0.0 –1.8 V	41.0 mA h g^−1^ / 0.06 A g^−1^	43.0 W h Kg^−1^ / 63.0 W Kg^−1^	67.1% / 1000	[34]
	MnAl‐LDH//PTCDI	0.5 m aqueous (NH_4_)_2_SO_4_	0.0 –1.5 V	57.7 mA h g^−1^ / 0.1 A g^−1^	45.8 W h Kg^−1^ / 163.5 W Kg^−1^	92.1% / 100	[37]
	Ni‐APW//PNNI	1 m aqueous NH_4_AC	0.0 –1.8 V	44.4 mA h g^−1^ / 0.1 A g^−1^	68.7 W h Kg^−1^ / 383.8 W Kg^−1^	∼100% / 10000	[38]
	NiHCF@CNTs//poly (1,5‐NAPD)	9 m aqueous NH_4_AC	0.4 –1.5 V	143.0 mA h g^−1^ / 1.0 A g^−1^	31.8 W h Kg^−1^ / 2266.0 W Kg^−1^	88.5% / 500	[39]
	PTCDI//N‐CuHCF	5.8 m aqueous (NH_4_)_2_SO_4_	0.4 –2.0 V	48.2 mA h g^−1^ / 0.3 A g^−1^	31.0 W h Kg^−1^ / 443.0 W Kg^−1^	71.9% / 1000	[40]
Dual‐ion battery	NTP//Na‐PW	5:5 aqueous (NH_4_)_2_SO_4_ and Na_2_SO_4_	0.0 – 2.0 V	79.8 mA h g^−1^ / 0.1 A g^−1^	38.1 W h Kg^−1^ / 48.0 W Kg^−1^	52.6% / 600	[42]
	PI//PTMA	1 m aqueous (NH_4_)_2_SO_4_	0.0 –1.9 V	136.5 mA h g^−1^ / 0.5 A g^−1^	51.3 W h Kg^−1^ / 15.8 W Kg^−1^	86.4% / 10000	[43]
	PI/NDC/CNF// PTMA/CNF	1 m aqueous (NH_4_)_2_SO_4_	0.0 –1.9 V	136.7 mA h g^−1^ / 0.5A g^−1^	114.3 W h Kg^−1^ / 18.6 KW Kg^−1^	71.7% / 2000	[44]

### 2.1 Rocking‐Chair Configuration

**FIGURE 3:**
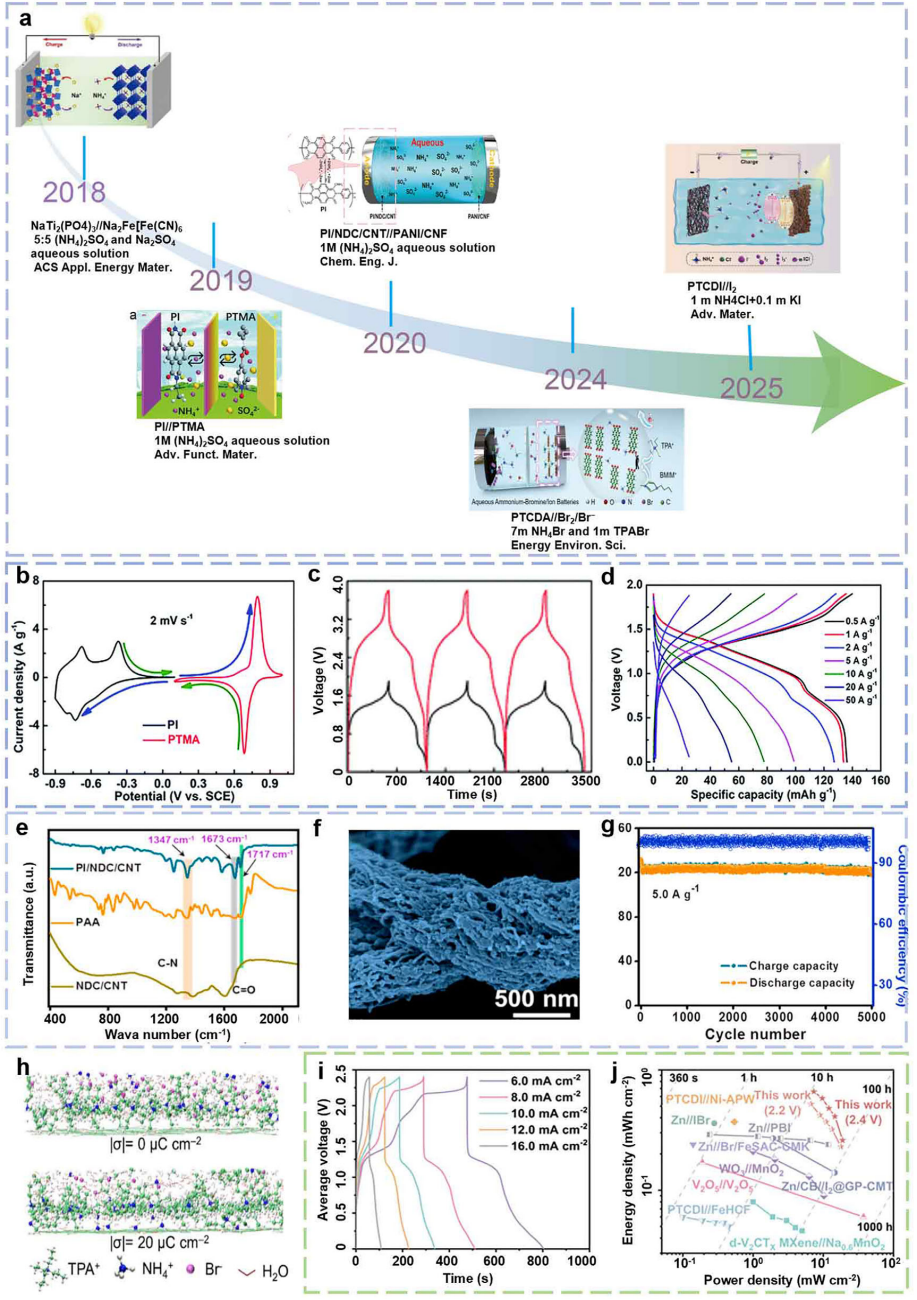
(a) Development timeline of NH_4_^+^‐based DIBs. (b) Comparison of CV curves of PI anode and PTMA cathode. (c) GCD profiles of individual device and two devices in series at 1 A g^−1^. (d) Rate performances of PI//PTMA battery at various current densities [43] (Copyright 2019, RSC). (e) FTIR of the PI/NDC/CNT anode. (f) SEM of the PI/NDC/CNT anode. (g) Cycling stability and coulombic efficiency (CE) of the PI/NDC/CNT anode at a current density of 5.0 A g^−1^ [44] (Copyright 2020, Elsevier). (h) MD simulation snapshots of PTCDA interfacial structure in 7 m NH_4_Br+1 m TPABr [45] (Copyright 2024, Elsevier). (i) GCD profiles of the PTCDI//I_2_ collected at different voltages. (j) Ragone plots of PTCDI//I_2_ with other reported storage devices ^[^ ^21^ ^]^ (Copyright 2025, Wiley‐VCH).

**FIGURE 4:**
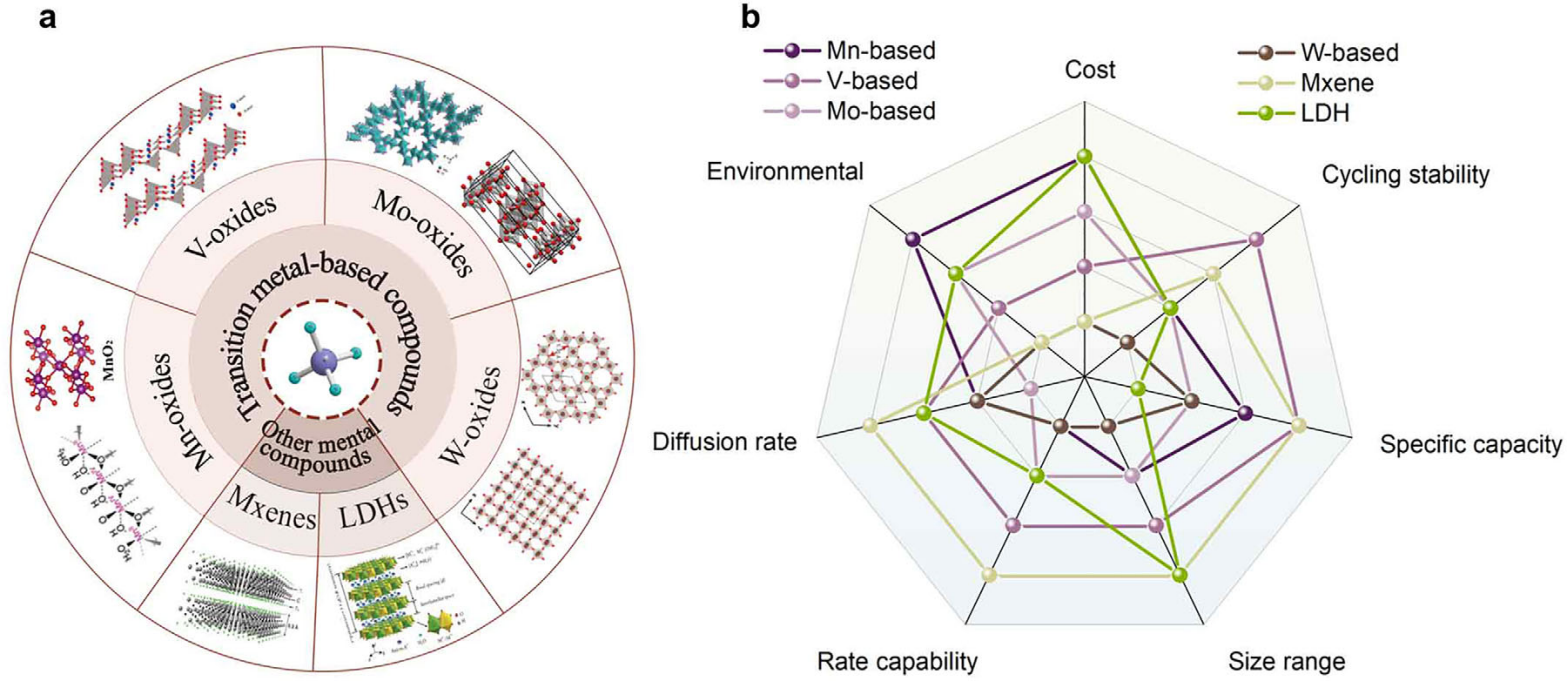
(a) Major categories of TMCs. (b) Radar plot evaluating representative TMCs across multiple performance metrics (rating:1–5).

The rocking‐chair configuration has been the most extensively studied design for NH_4_^+^‐based batteries, drawing widespread attention over the past decade. In such systems, reversible NH_4_^+^ shuttling is achieved through host–guest electrochemistry, with either the anode or cathode serving as the NH_4_^+^ reservoir during charge and discharge (Figure 2a). This design ensures unidirectional cation transport and stable interfacial dynamics, which are vital for achieving high reversibility and low polarization. The concept of NH_4_^+^‐based batteries dates back to 1982, when Toshima et al. first reported the unique electrochemical interaction between NH_4_^+^ and Prussian blue, laying the foundation for future research [33]. However, it was not until 2017 that Wu et al. first reported an aqueous rocking‐chair ammonium‐ion battery, employing (NH_4_)_1.47_Ni[Fe(CN)_6_]_0.88_ (Ni‐APW) as the anode (Figure 2b,c) and 3,4,9,10‐perylene‐bis(dicarboximide) (PTCDI) as the cathode [34]. This pioneering work established the feasibility of using NH_4_^+^ as a reversible charge carrier in aqueous media, thereby igniting renewed research interest in this emerging field.

Since then, extensive efforts have been devoted to optimizing NH_4_^+^ storage mechanisms and developing structurally compatible electrode materials. In 2021, Xie et al. investigated a rocking‐chair type ammonium‐ion battery utilizing a PBAs cathode and a PTCDI anode, revealing the crucial role of hydrogen bonding in the PBAs material (Figure 2d) [35]. The open framework and rigid Fe–CN–Fe network of PBAs enable fast ionic diffusion and superior structural reversibility [36]. However, their specific capacities remain inherently constrained by single‐electron redox activity and limited redox‐active sites, underscoring the need for more versatile TMC hosts, such as transition metal oxides, sulfides, carbides, and nitrides, to achieve higher capacity and stability in rocking‐chair AAIBs. In 2022, Hu et al. pioneered the use of manganese–aluminium layered double hydroxide (Mn_3_Al‐LDH) in AAIBs, which enables stable energy storage through a synergistic mechanism combining redox activity and hydrogen bonding. The Mn_3_Al‐LDH electrode delivers a high discharge capacity of 183.7 mA h g^−1^ at 0.1 A g^−1^, and when paired with a PTCDI anode in a battery, achieves an energy density of 45.8 W h kg^−1^, surpassing most previously reported systems (Figure 2e) [37].

Despite these significant advances, several critical challenges in long‐term cycling stability must be addressed before AAIBs can achieve widespread practical deployment. The stability issues mainly arise from the relatively large ionic radius of NH_4_^+^, which induces significant structural strain and phase degradation in the electrode during repeated ion insertion and extraction. In 2023, to further extend cycle life, Cao et al. returned to the Ni‐APW cathode but introduced the carbonyl‐rich organic anode 1,4,5,8‐naphthalenetetracarboxylic anhydride naphthylamine (PNNI), the carbonyl groups reversibly ligate NH_4_^+^ through hydrogen‐bond‐like interactions, delivering virtually zero capacity fade over 10 000 cycles at 1.0 A g^−1^ with negligible capacity decay (Figure 2f,g) [38]. At the same time, electrolyte instability at high operating voltages represents another key bottleneck. In response, researchers have been actively exploring innovative electrolyte formulations to enhance the electrochemical and thermal performance of AAIBs. For instance, Dong et al. developed a wide‐temperature‐range electrolyte system for AAIBs, utilizing low‐temperature‐compatible solvents (e.g., acetonitrile (AN)/propylene carbonate (PC)) and film‐forming additives (e.g., FEC) to suppress electrolyte freezing at −40°C, while optimizing NH_4_PF_6_ salt concentration to enhance high‐temperature stability. As a result, the battery operates stably across an unprecedented temperature range of −40°C to 80°C (Figure 2h,i), overcoming traditional thermal constraints [39].

Building on this progress, Alberto et al. proposed a high‐concentration electrolyte based on 5.8 m (NH_4_)_2_SO_4_ electrolytes in 2022. By significantly reducing the amount of free water and tailoring the NH_4_^+^ solvation structure, this electrolyte design expands the stable voltage window from ∼1.5 V to over 2.5 V (Figure 2j). The system exhibits exceptional cycling stability, retaining more than 90% of its initial capacity after 1000 cycles at 0.3A g^−1^, markedly superior to conventional low‐concentration systems, which typically retain only ∼70% capacity under similar conditions (Figure 2k) [40]. More recently, Sun et al. introduced a novel aqueous NH_4_^+^‐based battery configuration using an ammonium acetate (NH_4_Ac) electrolyte and V_2_CT*_x_* MXene electrodes. The NH_4_Ac electrolyte offers a near‐neutral environment (pH≈6.5), effectively mitigating electrode corrosion while enabling efficient ion transport. Theoretical simulations further revealed the presence of reversible electron transfer reactions involving [NH_4_^+^(HAc)_3_]‐O coordination bonds, which contribute to the system's exceptional NH_4_^+^ storage capability [41].

### 2.2 Dual‐Ion Batteries (DIBs) Configuration

The emergence of DIBs, characterized by their high discharge voltages, offers a promising route toward higher energy density. Unlike conventional rocking‐chair batteries, which rely solely on cation intercalation and deintercalation, DIBs employ both cations and anions as charge carriers. During charging, cations are stored in the anode while anions are simultaneously inserted into the cathode; while upon discharge, both species are released back into the electrolyte. This mechanism endows the electrolyte with a dual role: it serves not only as an ion‐conducting medium but also as the primary reservoir of electrochemically active species. As a result, salt concentration becomes a critical parameter in optimizing the performance of DIBs, directly influencing key metrics such as ionic conductivity, cycling stability, and energy efficiency. The electrolyte must therefore balance high ionic conductivity with a wide electrochemical stability window to sustain operation at elevated voltages without decomposition.

To elucidate the fundamental principles and evolution of this technology, we present a brief overview of the developmental history of DIBs, as illustrated in Figure 3a. In 2018, Ji et al. first proposed a NH_4_^+^‐based DIB in which Na_2_Fe[Fe(CN)_6_] (Na‐PW) hosts NH_4_^+^ and NaTi_2_(PO_4_)_3_ (NTP) accommodates Na^+^ during operation. This new system demonstrated promising capacity and cycle‐life performance [42]. In 2019, Zhang et al. developed an all‐organic NH_4_^+^‐based DIBs employing a p‐type polyimide (PTMA) as cathode and a n‐type polyimide (PI) as anode, utilizing 1 m ammonium sulfate aqueous electrolyte [43]. The cell delivered an operating voltage of 1.9 V and an energy density of 51.3 W h kg^−1^ (Figure 3b–d). Building on this work, Liu et al. demonstrated a high‐performance all‐organic aqueous NH_4_^+^‐based DIBs in 2020 by designing highly porous polyimide‐based nanofiber composite anodes (PI/NDC/CNT). This innovation effectively addressed the limitations of conventional anodes, including low capacity and short cycle life. Moreover, the study revealed the redox storage mechanism of NH_4_^+^ involving organic carbonyl and imine groups (Figure 3e–g) [44].

More recently, Liu et al. employed halide anions, which are inexpensive and electrochemically stable, to further increase energy density and broaden the electrochemical window. By leveraging the multi‐electron redox activity and high discharge plateau of bromide anions in combination with an ionic‐liquid‐functionalized electrolyte, they realized aqueous NH_4_^+^/Br DIBs that exhibit high voltage, long cycle life, and reliable operation. This system integrates a hydrophobic ionic liquid tetra propyl ammonium bromide (TPABr) with an NH_4_Br aqueous solution to form a biphasic electrolyte architecture (Figure 3j) [45]. Based on this, our team introduced a photo‐assisted mechanism coupled with an innovative device architecture to unlock a four‐electron redox pathway for iodide ions. This strategy enabled an ultrahigh voltage window of 2.4 V in an aqueous NH_4_^+^/I DIBs, using perylene tetracarboxylic diimide (PTCDI) as the anode and Co_3_O_4_‐TiO_2_ heterojunction as the photocathode (Figure 3i,j) [21]. Although significant progress has been made in DIBs systems over the past years, considerable challenges remain in further enhancing their cycling stability and practical energy density. Compared to well‐established rocking‐chair batteries, DIBs are still in their infancy. Continued exploration of reaction dynamics, interphase chemistry, and electrolyte engineering is essential to bridge the gap between laboratory‐scale prototypes and real‐world commercial applications.

## 3 Electrode Materials

Building upon the structural and mechanistic understanding of various AAIB configurations discussed above, the next critical factor determining device performance lies in the choice of electrode materials. The search for key materials that are both electrochemically reversible and chemically robust in aqueous media is essential for improving the overall performance of AAIBs. To date, several classes of host materials have garnered significant interest for NH_4_^+^ storage, including PBAs [33, 35], transition metal compounds (TMCs) [23, 37], organic electrodes [31, 38], and other emerging alternatives [46, 47]. Among these, TMCs stand out owing to their low cost, earth abundance, environmental compatibility, and structural robustness (Table 2). Figure 4a,b provides an overview of representative materials employed for NH_4_^+^ storage across various typical families, illustrating the diversity of host frameworks together with their respective advantages and shortcomings, while Table 3 compiles the corresponding electrochemical performance metrics of these exemplary TMCs.

**TABLE 2:** Summary of diffusion and capacity properties for various electrode materials.

	Strengths	Weaknesses	Environmental friendly
PBAs	High diffusion coefficient	Unsatisfied capacity	***
	high theoretical capacity	Low energy density	
	Inexpensive		
TMCs	Low toxicity	Lattice expansion	*****
	High capacity	Low electronic conductivity	
	High stability		
Organic materials	Lightweight	Complex fabrication	****
	High designability	Poor structural stability	
	High capacity		

**TABLE 3:** Comparison of performance in various TMCs for AAIBs.

	Host material	Electrolyte	Reference electrode	Working voltage	Capacity	Capacity retention	Ref
Mn‐based	δ‐MnO_2_	1 m aqueous NH_4_AC	SCE	0.0–0.8 V	176.0 mA h g^−1^@ 0.5 A g^−1^	94.7% / 10000	[53]
	α‐MnO_2_	1 m aqueous (NH_4_)_2_SO_4_	Ag/AgCl	0.0–1.6 V	219.0 mA h g^−1^@ 0.1 A g^−1^	95.4% / 10000	[57]
V‐based	V_2_O_5_·300H_2_O	0.5 m aqueous (NH_4_)_2_SO_4_	Ag/AgCl	−0.2 to 0.8 V	103.0 mA h g^−1^@ 0.1 A g^−1^	80.1% / 30000	[68]
	PANI/VO*_x_*	0.5 m aqueous (NH_4_)_2_SO_4_	SCE	−0.5 to 0.9 V	307.0 mA h g^−1^@ 0.5 A g^−1^	42.3% / 100	[69]
	NH_4_V_4_O_10_	1 m aqueous (NH_4_)_2_SO_4_	Ag/AgCl	0.0–1.0 V	167.0 mA h g^−1^ @ 0.1 A g^−1^	82.5% / 100	[73]
	VO_2_	1 m aqueous NH_4_AC	SCE	−0.9 to 0.0 V	385.0 mA h g^−1^@ 0.5 A g^−1^	56.1% / 1000	[82]
Mo‐based	*α*‐MoO_3_/C	1 m (NH_4_)_2_SO_4_ / PVA	Ag/AgCl	−0.6 to 0.6 V	158.2 mA h g^−1^ @ 1 A g^−1^	92.7% / 5000	[90]
	*h*‐MoO_3_	1 m aqueous NH_4_Cl	SCE	−0.5 to 0.8 V	114.9 mA h g^−1^ @ 1 C	94.1% / 100 000	[23]
	MoS_2_@PANI	1 m aqueous NH_4_Cl	Ag/AgCl	−0.6 to 0.4 V	452.0 F g^−1^ @1 A g^−1^	86.3% / 5000	[98]
W‐based	m‐WO_3_	0.5 m (NH_4_)_2_SO_4_	Ag/AgCl	−1.0 to 0.8 V	150.6 mA h g^−1^ @ 0.1 A g^−1^	86.6% / 50	[102]
	h‐WO_3_	1 m aqueous (NH_4_)_2_SO_4_	Ag/AgCl	−1.2 to 0.8 V	82.1 mA h g^−1^ @ 1 A g^−1^	67.9% / 100 000	[105]
Other TMCs	NH_4_V_4_O_10_@MXene	1 m aqueous (NH_4_)_2_SO_4_	Ag/AgCl	0.0–1.0 V	229.0 mF cm^−2^ @ 1 mA cm^−2^	98.1% / 5000	[76]
	MnAl‐LDH	0.5 m aqueous (NH_4_)_2_SO_4_	Ag/AgCl	−0.2 to 1.0 V	183.8 mA h g^−1^ @ 0.1A g^−1^	81.1% / 400	[37]
	E‐CoNi‐LDH	1 m aqueous NH_4_AC	SCE	0.0–0.9 V	202.4 mA h g^−1^ @ 0.6A g^−1^	72.1% / 10000	[115]

From a mechanistic perspective, the essential distinction in NH_4_^+^ storage among TMCs arises from how hydrogen bonding participates in ion transport and how diffusion pathways are geometrically confined. These two factors fundamentally differentiate between layered and tunnel‐structured materials. In layered TMCs, NH_4_^+^ migration is governed by a dynamic hydrogen bonding‐mediated mechanism. The ion continuously forms and breaks hydrogen bonds with adjacent oxygen atoms within flexible interlayers, enabling quasi‐two‐dimensional transport with low activation barriers. Such reconfigurable hydrogen bonding minimizes steric hindrance and allows local lattice relaxation, which is favorable for fast kinetics but often compromises long‐term structural stability. In contrast, tunnel‐structured TMCs accommodate NH_4_^+^ through direct, one‐dimensional diffusion along rigid crystalline channels. In these materials, hydrogen bonding plays a limited and largely static role, while ion transport is primarily dictated by steric confinement within well‐defined tunnels. Although this mechanism suppresses interlayer breathing and enhances structural robustness, the restricted diffusion dimensionality often leads to higher migration barriers and rate limitations. These mechanistic differences explain the distinct electrochemical behaviours observed in layered versus tunnel‐type hosts, including differences in rate capability, reversibility, and cycling stability. On this basis, the following subsections discuss how specific TMC families exploit or mitigate these mechanistic constraints to optimize NH_4_^+^ storage performance.

Although TMCs store NH_4_^+^ through a broadly shared “layer/tunnel‐hydrogen‐bond” insertion mechanism, their divergent crystal architectures and redox chemistries give rise to distinct performance regimes, each excelling in certain metrics while faltering in others. Manganese‐based oxides represent the most cost‐effective option, leveraging earth‐abundant Mn and the Mn^3+^/Mn^4+^ redox couple to deliver high specific capacity in both tunnels and interlayered configurations. Yet they are fundamentally limited by Mn dissolution and poor electronic conductivity, resulting in rapid fade and sluggish kinetics. In contrast, vanadium‐based oxides achieve among the highest theoretical capacities (>300 mAh g^−1^) by exploiting multi‐electron V^5+^/V^4+^/V^3+^ redox processes within polymer‐ or water‐pillared interlayers. However, this performance comes at the cost of vanadium's toxicity and structural instability during prolonged cycling. Molybdenum‐based compounds represent a more balanced compromise in NH_4_^+^ storage. Their rigid corner‐sharing MoO_6_ octahedral tunnel frameworks provide well‐defined one‐dimensional diffusion pathways that minimize activation barriers for NH_4_^+^ migration, thereby enabling highly reversible insertion/extraction and outstanding long‐term cycling stability. Yet their relatively narrow operational voltage window and susceptibility to parasitic side reactions limit achievable energy density. Tungsten‐based oxides push performance further in hybrid devices, where water‐stabilized open‐framework crystal structures support dual hydrogen bonding and yield ultrahigh areal capacity, wide voltage operation, and outstanding retention in NH_4_^+^‐based hybrid devices. Nevertheless, WO_3_ remains intrinsically constrained by high NH_4_^+^ diffusion barriers and significant lattice strain, which promote structural degradation during extended cycling.

Finally, emerging 2D TMCs such as MXenes and LDHs leverage multiple redox‐active metal centers to achieve high capacity. However, their practical implementation is impeded by high interfacial resistance and severe metal leaching, challenges that are significantly less pronounced in dense oxide frameworks. The following subsections provide a detailed account of the structural features and NH_4_^+^ storage behavior of each material class.

### 3.1 Manganese‐Based Oxides and Their NH_4_^+^ Storage Mechanism

**FIGURE 5:**
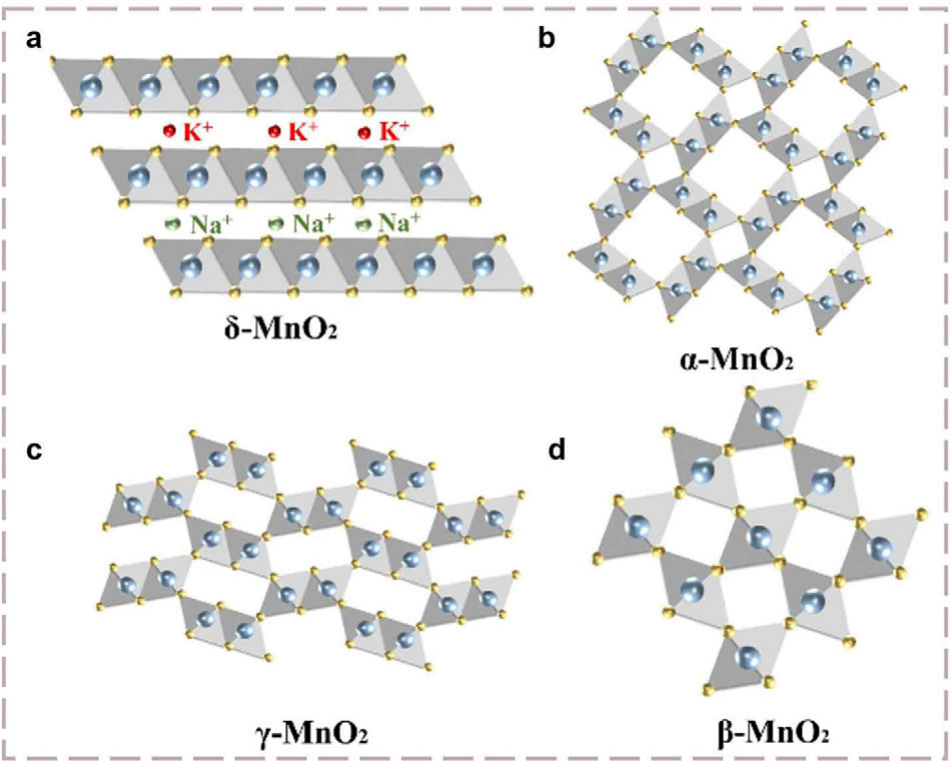
Crystal structure diagrams of MnO_2_ [52] (Copyright 2023, Wiley‐VCH).

**FIGURE 6:**
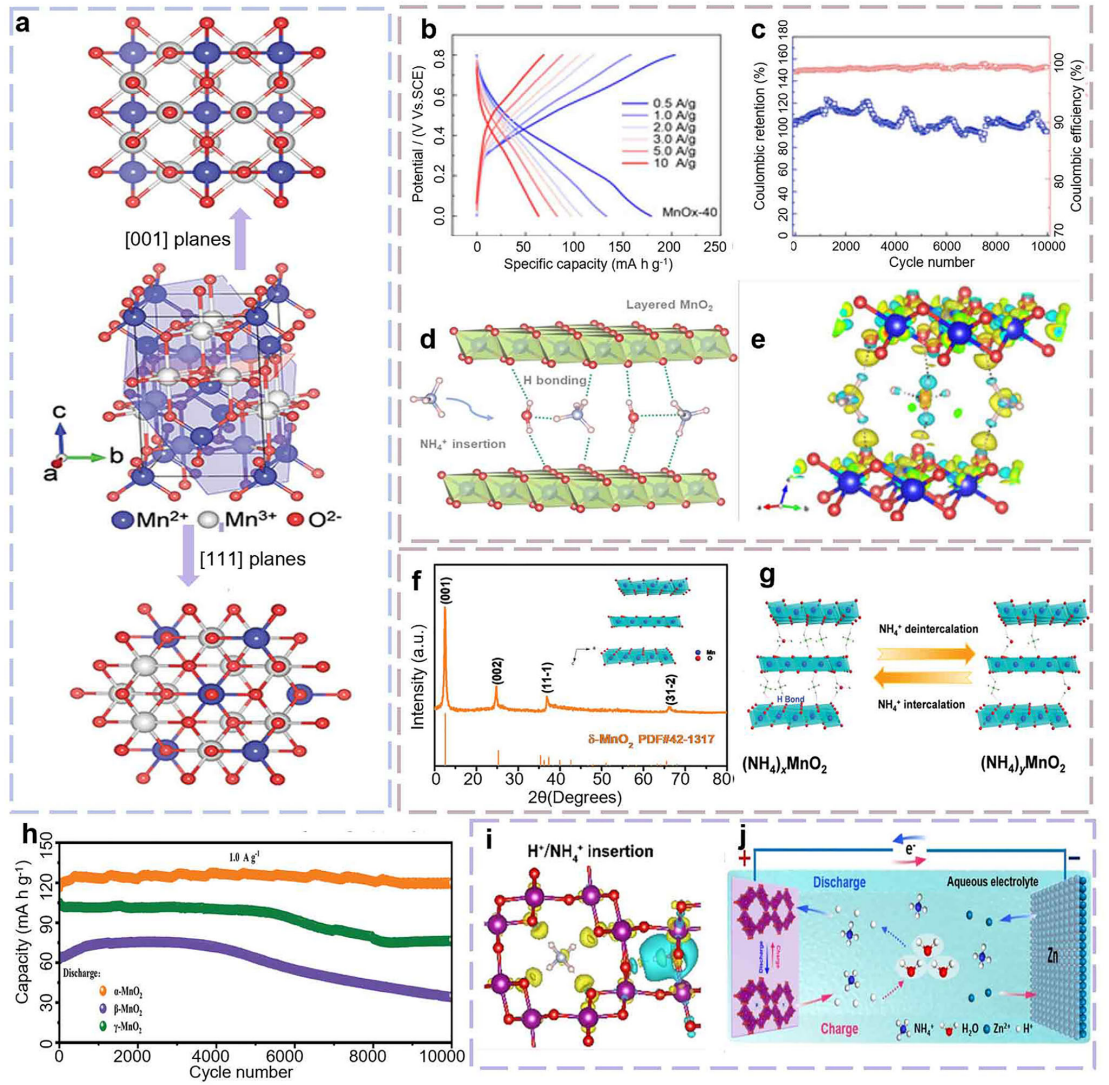
(a) Crystal structure diagrams of Mn_3_O_4_ nanosheets. The blue sites represent Mn^2+^ atoms, the white sites represent Mn^3+^ atoms, and the red sites represent O^2−^ atoms. The red shadow represents the (001) lattice plane, while the blue shadow represents the (111) lattice plane ^[^ ^49^ ^]^ (Copyright 2022, Wiley‐VCH). (b) Cycling stability and CE of MnO_x−40_ at the current density of 5 A g^−1^. (c) GCD profiles of MnO*_x_*_−40_ at different current densities. (d) Schematic diagram of NH_4_^+^ transport in layered MnO_2_. (e) Diagram of charge density difference of NH_4_‐inserted layered MnO_2_. Blue iso‐surfaces represent charge accumulation (+0.005 e Å^−3^), and yellow iso‐surfaces represent charge depletion (−0.005 e Å^−3^) [53] (Copyright 2023, Wiley‐VCH). (f) XRD pattern of the *δ*‐MnO_2_. (g) Schematic illustration of the NH_4_^+^ storage mechanism in the layered *δ*‐MnO_2_ [24] (Copyright 2021, Wiley‐VCH). (h) Cycling stability of α‐, β‐, and γ‐MnO_2_ electrodes at 1.0 A g^−1^ [57] (Copyright 2024, Wiley‐VCH). (i) The charge density difference distribution after H^+^ and NH_4_^+^ insertion into the [1×1] and [2×2] tunnels of MnO_2_. (j) The mechanism of H^+^/NH_4_^+^ ions co‐insertion chemistry in the aqueous Zn/MnO_2_ battery system [58] (Copyright 2021, Wiley‐VCH).

Manganese‐based oxides have been extensively investigated as host materials for AAIBs, owing to their abundance, environmental compatibility, low cost, high operating voltages, and excellent theoretical capacities. As illustrated in Figures 5 and 6, these materials can adopt diverse structural frameworks, including spinel, layered, tunnel, and mixed‐phase architectures, which provide abundant diffusion channels and flexible coordination environments for NH_4_^+^ accommodation. Such structural versatility enables multiple charge‐storage pathways involving both intercalation and surface redox reactions, thereby governing their overall electrochemical behavior [48, 49].

Among these manganese‐based oxides, the spinel‐type phase, typically represented by Mn_3_O_4_ (Figure 6a), was among the earliest structures to receive attention because of its good electrochemical reversibility. The spinel structure accommodates multiple oxidation states of manganese, such as Mn^2+^, Mn^3+^, and Mn^4+^, which occupy distinct crystallographic sites within the lattice. Specifically, Mn^2+^ predominantly resides in the tetrahedral A‐sites, while Mn^3+^ is mainly located in the octahedral B‐sites. This distribution of manganese ions with different oxidation states contributes to the unique electrochemical properties of spinel‐type manganese oxides [49]. By distinguishing between A‐ and B‐type sub‐units within the cubic spinel lattice, the relationship between structural features and electrochemical performance becomes more apparent. For example, Mn^2+^ at the A‐type sub‐units and Mn^3+^ at the B‐type sub‐units play critical roles in facilitating ion transport and enhancing the overall stability of the material during charge–discharge cycles. However, despite their promising electrochemical properties in other systems, spinel‐type manganese oxides face notable challenges when applied to ammonium‐ion storage. Specifically, the limited lattice interstices in Mn_3_O_4_ struggle to accommodate the relatively large NH_4_^+^, often resulting in structural expansion or even lattice degradation during intercalation [28, 50]. These structural distortions compromise the reversibility of NH_4_^+^ intercalation and accelerate capacity decay, thereby limiting the long‐term stability and practical applicability of spinel‐type manganese oxides in AAIBs.

Recently, to overcome the steric hindrance that limits NH_4_^+^ intercalation in spinel Mn_3_O_4_, Gao et al. introduced cobalt and exploited the high‐entropy effect by synthesizing a (Cr_0.2_Mn_0.2_Fe_0.2_Co_0.2_Ni_0.2_)_3_O_4_ spinel oxide containing five equimolar transition metals [51]. This strategy effectively disrupts the conventional symmetric ion‐diffusion channels of the spinel lattice and creates a 3D diffusion network well‐suited for NH_4_^+^ transport. By tailoring the local structure, the approach mitigates the steric constraints imposed by the large NH_4_^+^ ion, thereby markedly enhancing its storage performance. Benefiting from their intrinsic structural advantages, layered manganese oxides have also emerged as promising host materials for NH_4_^+^ storage. These materials consist of edge‐sharing [MnO_6_] octahedra sheets via weak van der Waals forces or electrostatic interactions. These lamellar frameworks provide open pathways that facilitate rapid intercalation of water molecules, protons, and alkali or ammonium ions, supporting rapid ion diffusion and reversible redox reactions, offering a complementary strategy to spinel oxides for efficient NH_4_^+^ storage (Figure 5) [52].

In 2020, Song et al. reported the first investigation of NH_4_^+^ storage chemistry using layered manganese oxide (MnO*_x_*), significantly advancing the understanding of ammonium‐ion energy storage mechanisms in transition metal oxides [53]. In their work, the layered MnO*_x_* electrode exhibited an impressive specific capacity of 176.0 m Ah g^−1^ at a current density of 0.5 A g^−1^ and demonstrated exceptional cycling stability over 10 000 cycles in 0.5 m NH_4_Ac electrolyte, outperforming previously reported NH_4_^+^‐hosting materials (Figure 6b,c). These improvements indicate that the reversible insertion and reinsertion of NH_4_^+^ are accompanied by the formation and breaking of hydrogen bonds between NH_4_^+^ and the MnO*_x_* layers, providing both structural flexibility and electrochemical reversibility (Figure 6e).

Notably, NH_4_^+^ insertion/extraction in layered MnO_2_ relies on a hydrogen bonding‐mediated mechanism: the ion rapidly shuttles between adjacent oxygen atoms by forming and breaking H‐bonds, minimizing steric hindrance and enabling low‐barrier diffusion (Figure 6d,e). Building on this foundation, Zhou et al. demonstrated in 2021 that δ‐MnO_2_ with an expanded interlayer spacing of approximately 0.26 nm serves as a high‐performance cathode for NH_4_^+^ storage [24]. The widened interlayer channels provide a two‐dimensional ion transport pathway that facilitates rapid and reversible NH_4_^+^ (de)intercalation (Figure 6f,g). The NH_4_^+^ storage mechanism in δ‐MnO_2_ is governed not only by the physical confinement effect within the interlayer space but also by hydrogen bonding interactions between non‐metallic atoms in the oxide layers and the hydrogen atoms of NH_4_^+^. This synergistic hydrogen‐bonding network effectively prevents interlayer collapse during charge–discharge cycles, thereby maintaining long‐term cycling stability. Current research continues to focus on optimizing layered manganese‐based materials through strategic modifications such as oxygen vacancy engineering, interlayer expansion, and other structural modifications. These approaches enhance ionic conductivity, facilitate NH_4_^+^ diffusion kinetics, and improve structural stability, thereby boosting overall electrochemical performance in AAIBs [54, 55].

Tunnel‐type manganese oxides, along with layered manganese oxides and spinel‐type counterparts, have attracted increasing attention as NH_4_^+^ host materials. These oxides feature an open‐framework tunnel structure, which not only provides sufficient space for NH_4_^+^ accommodation but also ensures structural stability during repeated ion insertion and extraction (Figure 5). In contrast to the dynamic hydrogen bonding‐mediated mechanism observed in layered MnO_2_, where NH_4_^+^ shuttles between adjacent layers through continuous formation and rupture of hydrogen bonds, NH_4_^+^ insertion in tunnel‐type oxides proceeds via direct, one‐dimensional diffusion along rigid crystalline channels. During the charging process, NH_4_^+^ migrates through the electrolyte to the cathode surface and subsequently enters the interstitial or tunnel structures of the manganese oxide via ion diffusion channels. This process involves both electric field‐driven ion migration and solid‐state diffusion within the host material. Upon discharging, NH_4_^+^ migrates through the electrolyte to the cathode surface and subsequently enters the interstitial or tunnel structures of the manganese oxide via ion diffusion channels. Upon charging, NH_4_^+^ is deintercalated from the cathode and returns to the electrolyte, thereby completing a full charge–discharge cycle. The rational design of such tunnel architectures enables rapid ion insertion and extraction during electrochemical operation, significantly enhancing the battery's rate capability and cycling stability. More importantly, manganese oxides typically consist of edge‐sharing [MnO_6_] octahedra that assemble into one‐dimensional tetragonal (1×1) and (2×2) tunnel structures, which can be tailored to meet a range of electrochemical requirements [56]. By precisely engineering the size and geometry of these tunnels, ion diffusion pathways can be optimized to further improve overall battery performance.

For instance, Liu et al. systematically investigated the energy storage characteristics of α‐, β‐, and γ‐MnO_2_ polymorphs by regulating their tunnel structures. Their study revealed that the α‐MnO_2_ cathode material, featuring a unique 2 × 2 tunnel configuration, delivers a high discharge capacity of 219.0 mA h g^−1^ at a current density of 0.1 A g^−1^, along with excellent cycling stability‐retaining 95.4% of its initial capacity after 10 000 cycles at 1.0 A g^−1^ (Figure 6h), underscoring its potential as a promising cathode for high‐performance AAIBs [57]. Although the (1 × 1) tunnels of α‐MnO_2_ are too small to accommodate NH_4_^+^, smaller H^+^ can still be intercalated. Accordingly, Niu et al. proposed the co‐intercalation of non‐metallic ions (H^+^ and NH_4_^+^) into α‐MnO_2_ in Zn||MnO_2_ batteries, achieving both high cycling stability and enhanced capacity retention, thereby demonstrating the material's strong potential for NH_4_^+^ storage (Figure 6i,j) [58]. To enhance the electrochemical performance of tunnel‐type manganese oxides, researchers are pursuing targeted strategies such as cation/anion doping, surface coating, and framework stabilization. These approaches aim to improve electronic conductivity, stabilize the tunnel architecture during ion insertion, and facilitate faster NH_4_^+^ diffusion, critical factors for achieving high‐rate capability and long‐term cyclability in practical AAIBs.

Apart from the aforementioned types of manganese oxide, mixed‐framework manganese oxides, such as Todorokite‐type MnO_2_ and layered‐tunnel intergrowths, have emerged as promising candidates for ammonium‐ion storage due to their hierarchical architectures. These materials integrate the structural robustness of tunneled frameworks with the expanded interstitial spaces characteristic of layered phases, enabling efficient ion transport and enhanced stability [59]. These tunnel‐layered structures combine the structural stability of the tunnel framework with the advantage of enlarged pore sizes, enabling efficient ion transport and enhanced cycling stability. For instance, the 3 × 3 tunnel pores in Todorokite‐type MnO_2_ measure approximately 0.69 nm×0.69 nm, which is significantly larger than the 1 × 1 or 2 × 2 tunnels in *β*‐MnO_2_ and *α*‐MnO_2_ (approximately 0.46 nm and 0.69 nm, respectively). Such enlarged tunnel dimensions not only facilitate the efficient insertion and extraction of large multivalent cations such as Mg^2+^, Ca^2+^, and Al^3+^, but also enable the reversible transport of hydrated ions, thereby enhancing overall electrochemical performance [60, 61, 62].

Despite their potential, the controllable synthesis of mixed‐framework manganese oxides remains a significant challenge. The formation of the Todorokite phase typically requires harsh conditions, such as prolonged high‐temperature hydrothermal treatment or multi‐step ion‐exchange processes, which are energy‐intensive and difficult to scale. Moreover, due to subtle differences in nucleation kinetics and thermodynamic stability, Todorokite often crystallizes alongside competing MnO_2_ polymorphs‐including *α*‐MnO_2_, *β*‐MnO_2_, and birnessite‐resulting in phase impurities that compromise structural integrity and electrochemical performance. Concurrently, fundamental aspects of NH_4_^+^ storage within these architectures remain poorly understood. These include the distribution of storage sites within the tunnels, the dynamic intercalation mechanism, and the structural stability during cycling. To bridge these knowledge gaps, advanced in situ and operando characterization techniques‐such as neutron diffraction, synchrotron X‐ray diffraction, and solid‐state nuclear magnetic resonance (NMR)‐combined with first‐principles calculations, are essential for elucidating the structure–property relationships governing ammonium‐ion insertion.

Overall, manganese‐based oxides offer versatile crystal structures and multi‐valence chemistry, with layered or tunneled frameworks enabling efficient NH_4_^+^ diffusion and hydrogen‐bond‐assisted stabilization, resulting in high capacity and robust cycling performance. Moving forward, research should focus on addressing these challenges to fully exploit the potential of mixed‐framework manganese oxides for high‐performance AAIBs.

### 3.2 Vanadium‐Based Oxides and Their NH_4_^+^ Storage Mechanism

Vanadium oxides represent one of the most promising families of functional materials, widely recognized in the field of energy storage and conversion. Their appeal stems from several key factors: abundant natural reserves, second only to iron, titanium, and manganese among transition metals in the Earth's crust, multi‐electron redox behavior, and structural versatility. The tunability of vanadium's oxidation states arises from its [Ar]4s^2^d^3^ electronic configuration, which enables a wide range of oxidation states from +5 to −3, with +2, +3, +4, and +5 being the most prevalent [63, 64]. This enables the formation of single‐oxidation‐state compounds such as VO (+2), V_2_O_3_ (+3), VO_2_ (+4), and V_2_O_5_ (+5), as well as mixed‐valence derivatives. Furthermore, the wide variety of oxygen coordination geometries‐including octahedral, pentagonal bipyramidal, square pyramidal, and polyhedral motifs that share corners, edges, or faces‐further endows vanadium oxides with unique chemical and electrochemical properties [65, 66, 67].

**FIGURE 7:**
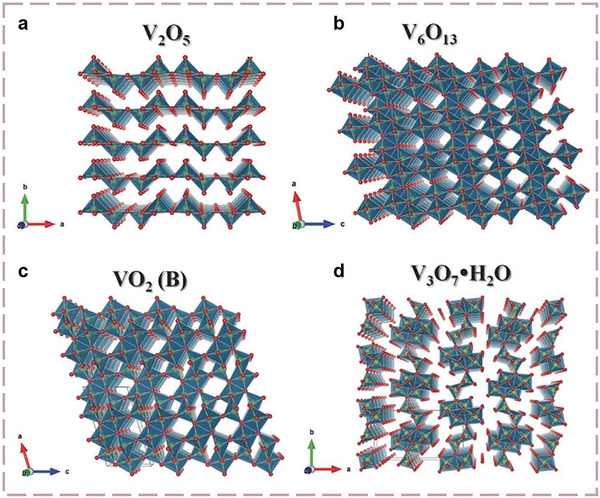
Crystal structure diagrams of vanadium‐based oxides [79] (Copyright 2017, Wiley‐VCH).

V_2_O_5_ is a prototypical vanadate oxide and one of the most extensively investigated cathode materials for NH_4_^+^‐based electrochemical energy storage. It features a layered structure composed of bilayers of edge‐ and corner‐sharing VO_6_ octahedra, which are stacked along the *c*‐axis and held together by weak van der Waals forces (Figure 7). Water molecules are intercalated within the interlayer galleries, serving to stabilize the structure and expand the interlayer spacing—typically to over 10 Å in hydrated phases—thereby creating spacious diffusion channels within the *a*–*b* plane. This open framework enables efficient and reversible intercalation/deintercalation of NH_4_^+^ ions (ionic radius ≈ 1.48 Å), while the ability of vanadium to undergo multi‐electron redox transitions (V^5+^/V^4+^ and V^4+^/V^3+^) contributes to high theoretical capacity.

**FIGURE 8:**
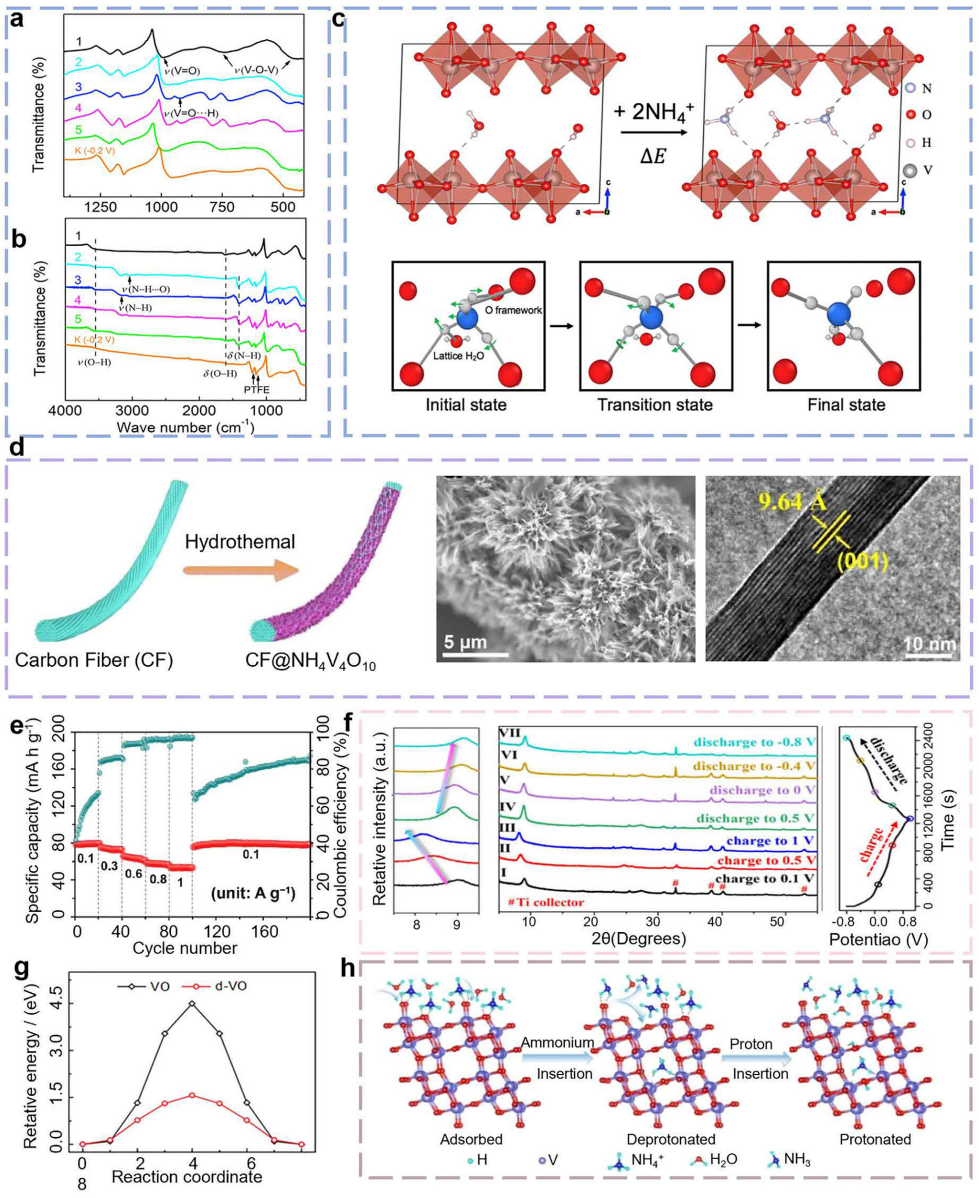
(a, b) Ex Situ FTIR Spectra of NH_4_^+^ into V_2_O_5_. (c) Schematic diagram of NH_4_^+^ transport in layered V_2_O_5_ [62] (Copyright 2019, Cell Press). (d) HRTEM of NH_4_V_4_O_10_. (e) Cycling stability and coulombic efficiency of NH_4_V_4_O_10_ [73] (Copyright 2020, Elsevier). (f) In situ XRD spectra of NH_4_^+^ into NVO [75] (Copyright 2022, Elsevier). (g) Diffusion activation energy of NH_4_^+^ along the VO_2_ tunnel [81] (Copyright 2022, Wiley‐VCH). (h) Schematic diagram of NH_4_^+^ transport in VO_2_ (B) [82] (Copyright 2023, Wiley‐VCH).

A key factor underpinning the excellent NH_4_^+^ storage performance in V_2_O_5_ is the formation of strong hydrogen‐bonding interactions between the inserted NH_4_^+^ and the oxygen atoms of the host lattice. In 2019, Sheng et al. reported a unique hydrogen‐bonding interaction between NH_4_^+^ and the interlayer hydroxyl groups of V_2_O_5_, forming a strong V═O···H─N network with a bond energy reaching 1.63 eV between NH_4_^+^ and V_2_O_5_ [22]. This interaction significantly reduces the diffusion barrier for NH_4_^+^, enabling fast ion kinetics. As a result, the V_2_O_5_ cathode delivered a high reversible specific capacity of 103.0 mA h g^−1^ at 0.1 A g^−1^, demonstrating exceptional cycling stability with only 20% capacity fade after 30 000 galvanostatic charge–discharge (GCD) cycles. The hydrogen bonding mechanism involves synergistic interactions, including electrostatic adsorption between oppositely charged species during discharge and directional N─H···O bonding with highly electronegative oxygen atoms. As a result, the NH_4_^+^‐V hydrogen bonds exhibit stronger binding affinity compared to certain ionic interactions (e.g., K^+^‐V_2_O_5_ ionic bonds). Moreover, upon intercalation into the interlayer galleries of V_2_O_5_, NH_4_^+^ forms additional hydrogen bonds with crystalline water molecules, as illustrated in Figure 8a–c [62]. This expanded interlayer structure enhances the exposure of active sites and widens ion transport channels, thereby improving both ion transport kinetics and charge storage performance.

In 2022, Mai et al. reported an innovative polyaniline (PANI)‐intercalated layered vanadium oxide composite (PANI/V_2_O_5_) as a cathode for AAIBs. The intercalation of PANI expanded the interlayer spacing from 4.3 Å to 15.5 Å, substantially reducing the NH_4_^+^ diffusion energy barrier. This structural engineering enabled a record specific capacity of 307.0 mA h g^−1^ at 0.5 A g^−1^ [69]. In a complementary study, Wang et al. also employed PANI intercalation to expand the interlayer spacing to 13.99 Å, achieving superior rate capability and cycling stability [70]. Notably, they demonstrated that the NH_4_^+^ storage mechanism in PANI‐intercalated V_2_O_5_ (PANI/VO*_x_*) is not a simple additive effect of the individual components (PANI and V_2_O_5_) but rather arises from synergistic host–guest interactions. Specifically, the π‐conjugated backbone of PANI enhances the electron density on the oxygen sites of the vanadium oxide framework, strengthening the hydrogen bonding between NH_4_^+^ and lattice oxygen (O^2−^). These interactions exhibit partial covalent‐like character, as supported by spectroscopic and computational analyses, leading to faster NH_4_^+^ transport kinetics and exceptional reversibility, as evidenced by ∼98% capacity retention after cycling at a high current density of 10 A g^−1^.

Building on this concept, Huang et al. replaced PANI with reduced graphene oxide (rGO) to fabricate a free‐standing VOH/rGO composite film [71]. The rGO similarly enlarges and stabilizes the interlayer spacing (∼10.2 Å), while also suppressing nanosheet restacking and buffering volume changes via its 3D conductive network and flexible scaffold. This architecture delivered outstanding electrochemical performance in ammonium‐ion supercapacitors, maintaining ∼93% of its initial capacity after more than 10 000 cycles using a PVA (polyvinyl alcohol)/NH_4_Cl gel electrolyte [72]. Beyond these composites, ammonium vanadate (e.g., NH_4_V_4_O_10_ [73], NH_4_V_3_O_8_·2.9H_2_O [74], (NH_4_)_2_V_10_O_25_·8H_2_O [75]) has been reported recently for NH_4_^+^ storage with excellent electrochemical performance. In these materials, the intercalation of NH_4_^+^ into the vanadium‐oxygen layers results in a significant expansion of the interlayer spacing. For instance, the interlayer distance in NH_4_V_3_O_8_ reaches ∼9.8 Å, markedly exceeding that of pristine V_2_O_5_ (∼4.3Å), thereby facilitating rapid ion diffusion and enhancing structural stability during repeated cycling.

In 2020, Li et al. reported a sea urchin‐like NH_4_V_4_O_10_ anchored on carbon fibers as a promising cathode for AAIBs. The pre‐intercalated NH_4_^+^ in the V_4_O_10_ framework results in an expanded interlayer spacing of ∼9.5 Å, which not only facilitates rapid NH_4_^+^ diffusion but also exhibits excellent structural stability during cycling (Figure 8d). The resulting battery demonstrated a high specific capacity of ∼167 mA h g^−1^ at a current density of 0.1 A g^−1^, and a retained capacity of ∼54 mA h g^−1^ even at 1 A g^−1^, demonstrating excellent rate capability, ultralong cycle life, and high capacity retention (Figure 8e) [73]. In 2022, Wang et al. pioneered the exploration of NH_4_^+^ storage in a high‐performance hybrid supercapacitor (HSC) using a vanadium oxide framework (NH_4_)_2_V_10_O_25_·8H_2_O (NVO). This NVO nanostructure enabled reversible NH_4_^+^ insertion/extraction during charge–discharge processes (Figure 8f), attributed to its large interlayer spacing (∼10.6 Å) and structural compatibility with NH_4_^+^. When paired with an NH_4_Cl/PVA‐based gel electrolyte, the NVO cathode delivered an impressive specific capacitance of 339.0 F g^−1^ at 0.5 A g^−1^, demonstrating efficient charge storage kinetics [75]. However, such pre‐intercalated structures often suffer from structural degradation due to repeated volume expansion and contraction during long‐term cycling, posing a challenge to durability. To address this limitation and further enhance ion transport kinetics, Syam G. Krishnan et al. developed a composite electrode integrating ammonium vanadate (NH_4_V_4_O_10_) with conductive MXene (Ti_3_C_2_T*_x_*) nanosheets. The synergistic combination leverages the large interlayer spacing of NH_4_V_4_O_10_ and the metallic conductivity, hydrophilicity, and mechanical flexibility of MXene. This interlayered architecture facilitates rapid ion and electron transport, resulting in a high areal capacitance of 229.0 mF cm^−2^ at a current density of 1.0 mA cm^−2^, along with outstanding cycling stability, retaining approximately 98.1% of its initial capacitance after 5000 cycles [76].

Further advancing the performance frontier, Zhang et al. employed defect engineering to modulate the electronic structure and ion diffusion pathways in (NH_4_)_2_V_10_O_25_·8H_2_O [77]. By introducing oxygen vacancies under mild reduction conditions, they synthesized oxygen‐deficient NVO (denoted as OdM‐NVO), which exhibited enhanced electrical conductivity and increased active site availability. When assembled into a flexible quasi‐solid‐state HSC using a PTCDI anode (OdM‐NVO//PTCDI), the device achieved high areal capacitance and energy density, along with excellent mechanical flexibility and stable electrochemical performance under bending. These results highlight that pre‐intercalation, nanoarchitectural design, and defect control are complementary strategies for optimizing NH_4_^+^ storage in vanadium‐based systems. Beyond these strategies, heterostructure engineering has proven effective in modulating the interlayer spacing of host materials to enhance NH_4_^+^ storage performance. Further extending this paradigm, Du et al. fabricated a two‐dimensional VS_2_/VO*_x_* heterostructure through in situ electrochemical induction. The resulting heterointerface not only shortens the diffusion pathway for NH_4_^+^ but also provides abundant active sites, improved electrical conductivity, and enhanced structural stability, attributable to defect‐rich interfaces and synergistic interactions between the phases. As a result, the electrode achieves a high reversible capacity of 150.0 mAh g^−1^ and maintains robust cycling stability over 1000 cycles [78]. These results highlight interlayer engineering as a promising and versatile strategy for boosting NH_4_^+^ storage in layered materials for ammonium‐ion batteries.

Other vanadium oxides, including V_6_O_13_, V_3_O_7_·H_2_O, VO_2_ (B), and V_2_O_3_, also possess energy storage capability due to their unique structural characteristics [79, 80]. Among these, VO_2_ has recently garnered significant research interest. As a tunnel‐type vanadate oxide, VO_2_ features a bilayer framework composed of edge‐sharing VO_6_ octahedra, which creates open diffusion channels with a cross‐sectional area of approximately 8.2 Å^2^. These spacious tunnels provide efficient pathways for NH_4_^+^ intercalation and deintercalation, while favourable hydrogen bonding interactions between NH_4_^+^ and the oxide lattice (V–O···H–N) further stabilize ion insertion and enhance electrochemical reversibility (Figure 7). In 2022, Dong et al. first demonstrated that the NH_4_^+^ storage performance of VO_2_ could be effectively regulated through defect engineering, providing new insights into the structural evolution of VO_2_ during ammonium ion insertion and extraction (Figure 8g) [81]. When evaluated as an anode in a 1 m (NH_4_)_4_SO_4_ electrolyte, defect‐engineered VO_2_ delivered a reversible capacity of approximately 200.0 mA h g^−1^ at a current density of 0.1 A g^−1^. Building on this foundation, Mai et al. introduced a conceptually distinct strategy in 2023 to further enhance NH_4_^+^ storage through interfacial chemistry in AAIBs. Their approach enables the co‐insertion and extraction of H_3_O^+^ and NH_4_^+^ into the 1×1 tunnels of VO_2_(B). During operation, NH_4_^+^ migrates from the cathode to the anode–electrolyte interface, where they undergo deprotonation to form H_3_O^+^, which subsequently intercalates into the VO_2_ (B) lattice. This dual‐ion mechanism significantly improves ion transport kinetics while alleviating the structural strain typically associated with the insertion of large NH_4_^+^ ions (Figure 8h). Consequently, the VO_2_(B) anode delivers a high specific capacity exceeding 300.0 mA h g^−1^ with ultrafast charge storage kinetics [82]. These results highlight the potential of vanadium‐based oxides as high‐performance NH_4_^+^ hosts and set the stage for exploring other transition metal oxides.

### 3.3 Molybdenum‐Based Compounds and Their NH_4_^+^ Storage Mechanism

Molybdenum oxide exhibits rich redox chemistry, stabilizing in multiple oxidation states that give rise to a range of stoichiometric and non‐stoichiometric compounds. These span from fully oxidized molybdenum trioxide (MoO_3_)—a wide‐bandgap semiconductor (>2.7 eV)—through sub‐stoichiometric phases such as MoO_3−_ *_x_* (2< *x* < 3), to semi‐metallic MoO_2_ [83], which possesses a significantly reduced bandgap. The introduction of oxygen vacancies drives the reduction of Mo^6+^ to Mo^5+^ and further to Mo^4+^, enabling precise control over electronic carrier concentration and band structure. This tunability allows for deliberate engineering of the material's electronic properties through modulation of crystal phase, morphology, oxygen vacancy density, or heteroatom doping [84]. Owing to this chemical and functional versatility, molybdenum oxides find application across diverse fields, including catalysis, energy storage, sensing, electronics, antibacterial coatings, and agriculture [85].

**FIGURE 9:**
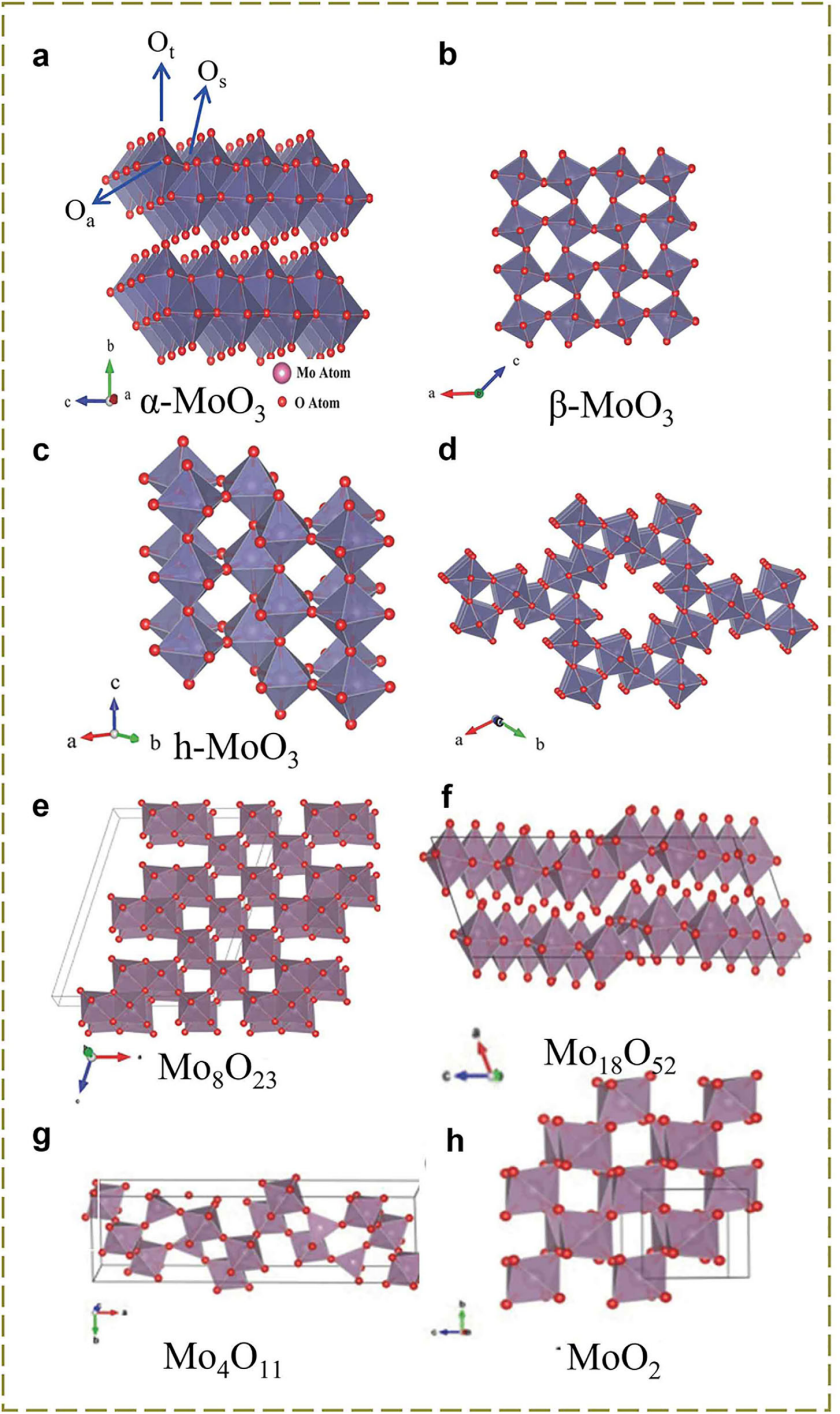
Crystal structure diagrams of various molybdenum‐based compounds [83] (Copyright 2017, Wiley‐VCH).

MoO_3_ is the most representative molybdenum‐based oxide and crystallizes in three polymorphic forms: orthorhombic *α*‐MoO_3_, monoclinic *β*‐MoO_3_, and hexagonal *h*‐MoO_3_. Under ambient conditions, *α*‐MoO_3_ is the most thermodynamically stable form (Figure 9a–d). It comprises distorted MoO_6_ octahedra that share corners along the *a*‐axis ([100] direction) and edges along the *c*‐axis ([001] direction), forming a layered structure [86]. These layers stack along the *b*‐axis ([010] direction) via weak van der Waals interactions, resulting in a well‐defined two‐dimensional architecture with an interlayer spacing of approximately 1.4 nm. Intralayer bonding is predominantly covalent and ionic in character (Figure 9a) [85, 87]. This layered motif resembles that of V_2_O_5_; however, differences in metal–oxygen bond configurations and strengths lead to distinct structural anisotropies and mechanical response. Within a single *α*‐MoO_3_ layer, three non‐equivalent oxygen sites exist: terminal oxygen (O_1_), doubly bridging oxygen (O_2_), and triply bridging oxygen (O_3_). Each distorted MoO_6_ octahedron consists of a molybdenum atom bonded to one O_1_ atom, two O_2_ atoms, and three O_3_ atoms. This layered structure endows *α*‐MoO_3_ with distinctive physicochemical properties, including anisotropic charge transport and tunable interlayer reactivity [88, 89]. Zhi et al. were the first to propose layered *α*‐MoO_3_ as an anode material for ammonium‐ion storage. However, its relatively narrow interlayer spacing and restricted structural openness impede efficient NH_4_^+^ diffusion, resulting in limited specific capacity and poor rate performance.

**FIGURE 10:**
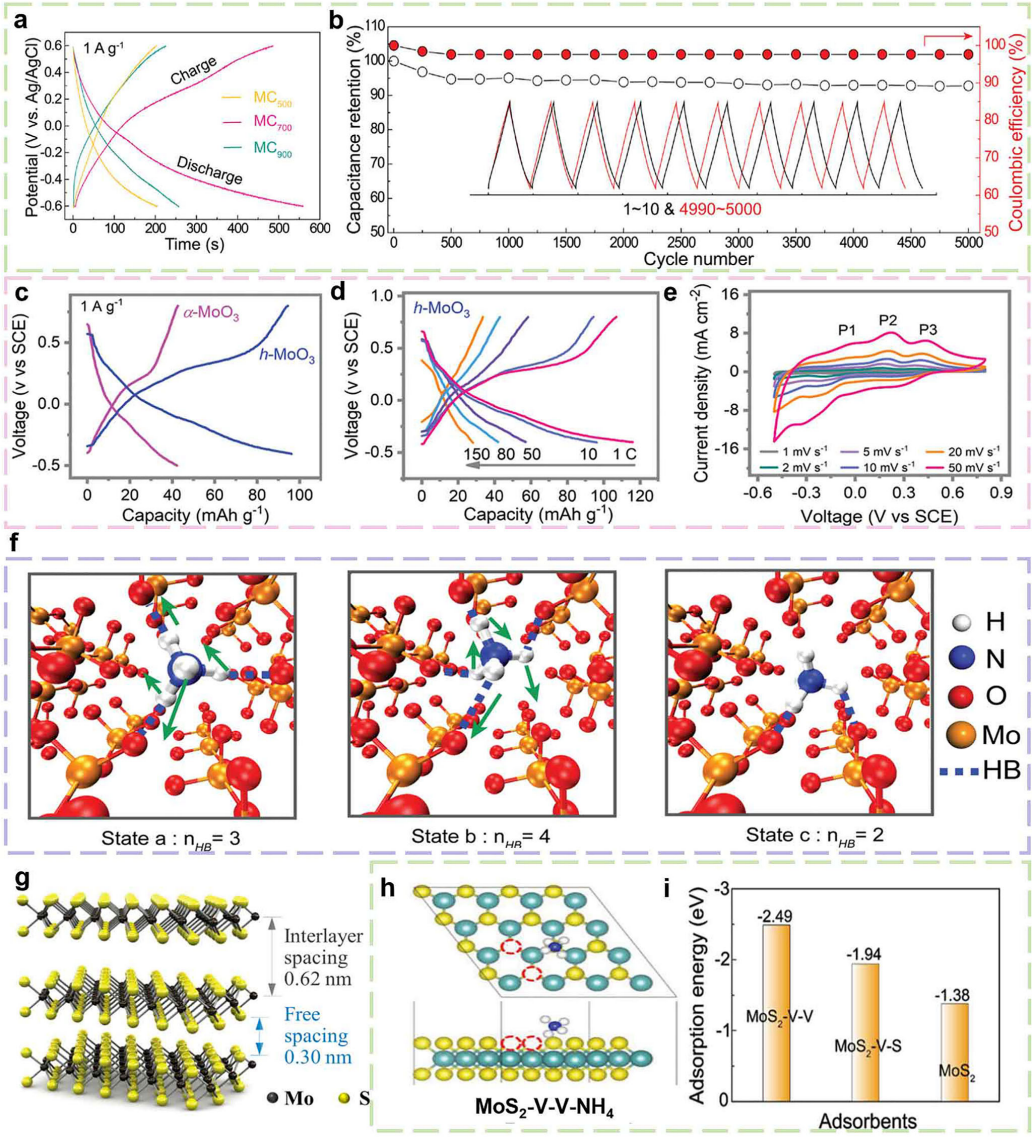
(a) GCD profiles of MoO_3_@C composite at a current density of 1 A g^−1^. (b) Cycling performance and CE of MoO_3_@C_700_ at a current density of 20 A g^−1^ [90] (Copyright 2023, Wiley‐VCH). (c) GCD profiles of *h*‐MoO_3_ and *α*‐MoO_3_ electrodes for NH_4_^+^ at a current density of 1 A g^−1^. (d) Rate performance of *h*‐MoO_3_ at different current densities. (e) CV curves of *h*‐MoO_3_ at different scan rates. (f) Schematic illustration of the three‐step NH_4_^+^ diffusion mechanism, showing hydrogen atom migration via green arrows [23] (Copyright 2020, Wiley‐VCH). (g) Crystal structure diagrams of MoS_2_ [97] (Copyright 2017, ACS). (h) Optimized structure of NH_4_^+^ adsorbed on double‐Sv (sulfur vacancies) MoS_2_. (i) Calculated adsorption energy of NH_4_^+^ on double‐Sv MoS_2_, single‐Sv MoS_2_ and pristine MoS_2_ [98] (Copyright 2023, Wiley‐VCH).

Building on these insights, Dai et al. reported a significant advancement in 2022 through the synthesis of MoO_3_ nanostructures with abundant oxygen vacancies, which were further coated with a conductive carbon layer to form a MoO_3_@C composite. This architecture substantially enhances the material's intrinsic electronic conductivity and reduces charge transfer resistance by facilitating efficient electron transport at the electrode interface. The optimized composite electrode delivered an exceptional specific capacitance of 437.0 F g^−1^ (158.0 mA h g^−1^; 1592 mF cm^−2^) at a current density of 1 A g^−1^. Moreover, it exhibited excellent cycling stability, retaining 92.7% of its initial capacitance after 5000 cycles [90] (Figure 10 a,b). This work provides new design principles for electrode materials in small‐ion storage systems, demonstrating that high performance arises not only from expanded interlayer spacing but also from atomic‐scale engineering of the chemical environment to promote favourable ion‐surface interactions.

Based on this, Tang et al. shifted their focus from “within the lattice” to “surface channels.” In 2023, they introduced a phosphate ion‐assisted surface functionalization strategy to improve the ammonium‐ion storage performance of *α*‐MoO_3_ electrodes [91]. Their work revealed the crucial role of the surface oxygen coordination environment in modulating the solvation structure of NH_4_^+^ and the interfacial hydrogen‐bonding interactions. Based on this mechanistic insight, they developed a high‐performance aqueous ammonium‐ion hybrid supercapacitor. Meanwhile, Lin et al. eliminated long‐range order altogether, creating an amorphous “maze” of open nano‐channels that offer short, multidirectional diffusion shortcuts for hydrated NH_4_^+^. By electrodepositing amorphous molybdenum oxide, they achieved electrode gravimetric/areal capacities of 175.1 mA h g^−1^ and 130.2 mA h cm^−2^, respectively [92]. In addition to the thermodynamically stable *α*‐MoO_3_ phase, two metastable polymorphs, monoclinic *β*‐MoO_3_ and hexagonal *h*‐MoO_3_, also exhibit distinct structural features due to different arrangements of their MoO_6_ octahedra. In *β*‐MoO_3_, the MoO_6_ octahedra share corner oxygen atoms along the *c*‐axis and edge oxygen atoms along the *a*‐axis. In contrast, *h*‐MoO_3_ consists of zigzag chains of MoO_6_ octahedra. These chains are connected via corner‐sharing oxygen atoms along the *c*‐axis, forming a hexagonal tunnel structure [93].

Compared to the tetrahedral tunnels found in tunnel‐type manganese oxides, the hexagonal channels in molybdenum‐based oxides are significantly larger. This structural feature offers greater accessibility for cation insertion and improved ion transport kinetics. These spacious hexagonal channels serve as favourable host structures for NH_4_^+^ storage and contribute significantly to structural stability (Figure 9c,d). After Zhi et al. proposed the use of MoO_3_ for NH_4_^+^ storage, the research team activated *h*‐MoO_3_ for the first time and applied it to aqueous NH_4_^+^ storage. They compared the ammonium storage capabilities of *h*‐MoO_3_ with those of *α*‐MoO_3_ and *β*‐MoO_3_. The tunnel structure of *h*‐MoO_3_ demonstrated clearly superior electrochemical performance (Figure 10c). It delivered a specific capacity of 115.0 mA h g^−1^ at a current density of 1C and retained a capacity of 32.0 mA h g^−1^ even at an ultrahigh rate of 150 C (Figure 10d,e), indicating excellent rate capability. More impressively, it retained ∼94% of its capacity after 100 000 cycles and exhibited a high‐power density of 4170.1 W kg^−1^ at 150 C [23]. This outstanding performance is due to the ion rotation‐diffusion mechanism within the tunnels. The movement of NH_4_^+^ involves both rotational and translational motions. The interaction between NH_4_^+^ and oxygen atoms on the tunnel walls triggers rotational motion of the ions inside the tunnels. Following this rotation, NH_4_^+^ moves from one position to another through translational motion. This combined process facilitates efficient ion diffusion (Figure 10f). In addition, reversible formation and dissociation of hydrogen bonds between NH_4_^+^ and corner‐sharing oxygen atoms in the hexagonal tunnels contribute to stabilizing the insertion and extraction processes. The structural stability provided by these hexagonal tunnels enhances cycling stability [23, 94, 95].

Non‐stoichiometric molybdenum oxides (MoO_3−_ *_x_*, 2< *x* < 3) can form some well‐ defined suboxides with an average valence between 6 (MoO_3_) and 4 (MoO_2_). Among all suboxides, Mo_18_O_52_, Mo_8_O_23_, and Mo_4_O_11_ are the most common crystal structures. Their typical characteristics are manifested as periodically arranged edge‐shared MoO_6_ octahedral clusters. However, these oxygen‐deficient structures are limited by low charging efficiency and poor cycling stability. MoO_2_ exhibits a distorted rutile structure, containing two inequivalent oxygen coordination sites and three different Mo─O bond lengths, which leads to a symmetric change from the tetragonal phase to the monoclinic phase. MoO_2_ has excellent physicochemical properties, including low resistivity, high melting point, outstanding chemical stability, and efficient charge transport capability. These properties make it widely used in lithium‐ion anodes, supercapacitors, solid oxide fuel cells, and electrocatalysis. However, its application in ammonium ion storage is still limited, mainly due to the oxidation risk at high potentials, the high diffusion barrier caused by the asymmetric layered channels, and the rigid lattice that cannot adapt to the volume change caused by NH_4_^+^ intercalation. Despite these limitations, there is still the possibility of achieving ammonium ion storage in the future through structural design of carbon‐composite structures or lattice engineering regulation (Figure 9e–h) [84, 96].

Molybdenum disulfide (MoS_2_) is increasingly being explored as an alternative to MoO_3_ for NH_4_^+^ storage, primarily due to its larger interlayer spacing of 0.62 nm. As shown in Figure 10g, a MoS_2_ monolayer consists of a molybdenum (Mo) atom layer sandwiched between two sulfur atom layers. The saturated sulfur atoms on the basal plane (excluding the edges) not only maintain the chemical stability of bulk MoS_2_ but also stabilize individual MoS_2_ monolayers. Adjacent monolayers are held together by weak van der Waals forces, enabling easy exfoliation into two‐dimensional nanosheets [97]. In a recent study, Dai et al. developed a high‐performance aqueous HSCs based on a sulfide composite electrode of MoS_2_ and polyaniline (MoS_2_@PANI) as the NH_4_^+^ host. Compared with other metal ions, MoS_2_ exhibits low‐strain NH_4_^+^ intercalation behavior, which is attributed to the smallest hydrated radius and the second‐smallest mass of NH_4_^+^. It demonstrates excellent electrochemical performance, including a high specific capacitance of 450.0 F g^−^^1^, 86.3% cycling stability after 5000 cycles, and an assembled symmetric supercapacitor that achieves an energy density exceeding 60.0 W h kg^−1^ at a power density of 725.1 W kg^−1^. The study further demonstrates that the adsorption capacity of MoS_2_ for NH_4_^+^ increases progressively with sulfur‐vacancy concentration (Figure 10h,i) [98]. Building on this foundation, Xie et al. took a decisive step forward: by pre‐intercalating K^+^, they expanded the MoS_2_ interlayer spacing to 9.3 Å and introduced structural water to act as both a charge shield and an ion‐transport lubricant. This strategy not only preserves the low‐strain advantage demonstrated by Dai and for the first time enables universal storage of six cations (K^+^, Na^+,^ Li^+^, NH_4_^+^, Mg^2+^, and Al^3+^) in a single electrode material. When applied as an NH_4_^+^ host, the material delivered a discharge capacity of 50.7 mA h g^−1^ and a high CE of 94.1% [99].

More recently, Li et al. developed a MoS_2_‐based anode for aqueous asymmetric supercapacitors by anchoring MoS_2_ nanosheets onto titanium nitride‐coated carbon nanotube fibers (MoS_2_@TiN/CNTF). The nanosheets formed strongly bonded heterointerfaces with the substrate through Mo─S─Ti chemical bonds, which significantly accelerated charge transfer kinetics. The performance enhancement was attributed to the abundance of active sites and the synergistic effects arising from the array‐like heterostructure engineering. As a result, the MoS_2_@TiN/CNTF anode exhibited a high areal specific capacitance of 1102.5 mF cm^−2^ at a current density of 2 mA cm^−2^ [100].

### 3.4 Tungsten‐Based Oxides and Their NH_4_^+^ Storage Mechanism

**FIGURE 11:**
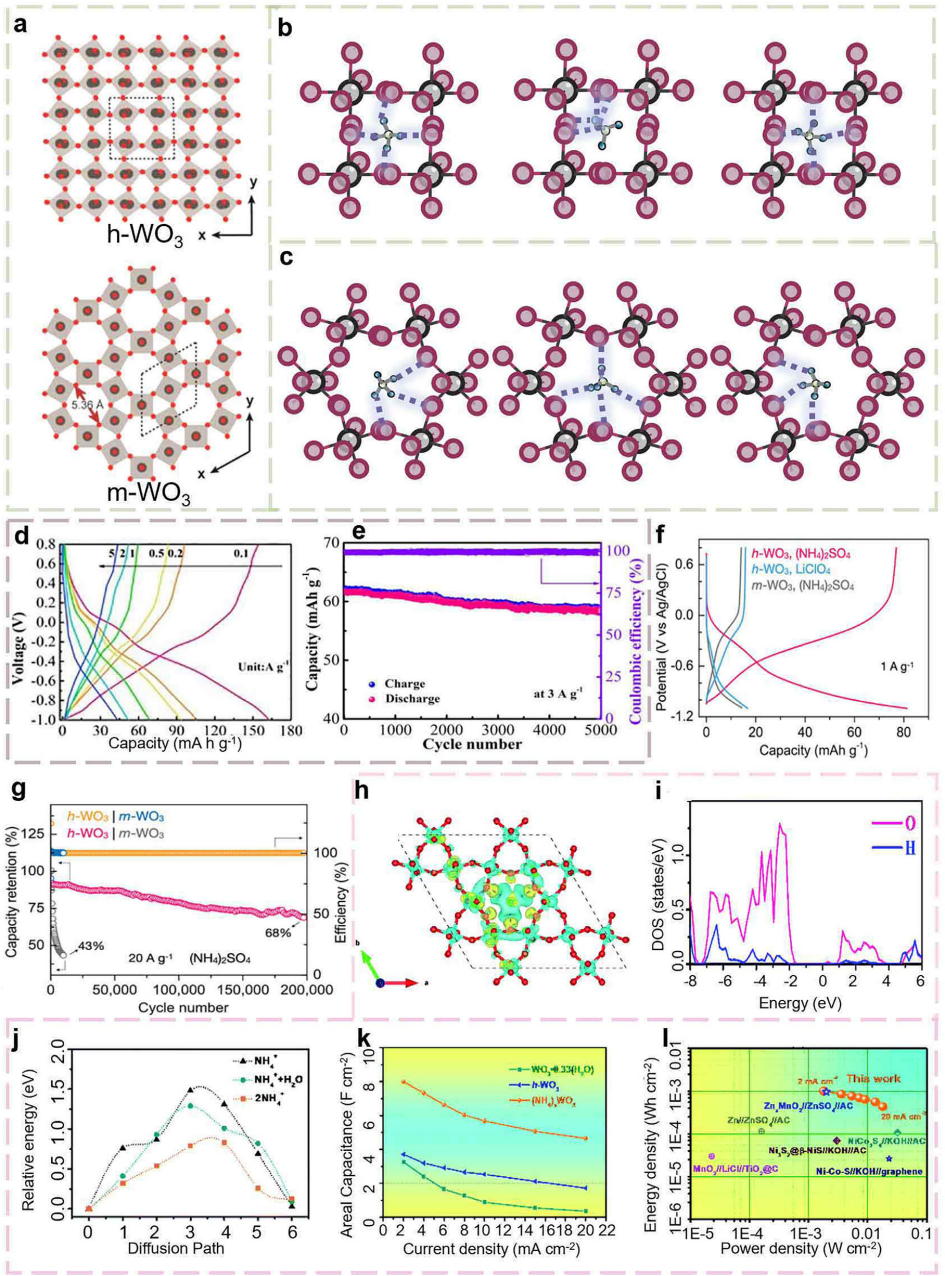
(a) Crystal structures of *h*‐WO_3_ and *m*‐WO_3_ viewed along the *c*‐axis [22] (Copyright 2024, Wiley‐VCH). Three stages of the NH_4_^+^ diffusion process in (b, c) *m*‐WO_3_ and *h*‐WO_3_. (d) GCD profiles of *m*‐WO_3_ at different current densities. (e) Long‐term cycling performance of the *γ*‐MnO_2_ // *m*‐WO_3_ full battery at 3.0 A g^−1^ [102] (Copyright 2023, Elsevier). (f) GCD profiles of *h*‐WO_3_ in 1 m (NH_4_)_2_SO_4_ or 1 m LiClO_4_ electrolytes, along with the GCD profiles of *m*‐WO_3_ in 1 m (NH_4_)_2_SO_4_. (g) Cycling performance of *h*‐WO_3_ and *m*‐WO_3_ in 1 m (NH_4_)_2_SO_4_ at 20 A g–^1^ [105] (Copyright 2022, Wiley‐VCH). (h) Charge density difference diagram of NH_4_^+^ intercalated *h*‐WO_3_. (i) The PDOS of hydrogen and oxygen that form hydrogen bonds. (j) Diffusion activation energy of NH_4_^+^ along the WO_3_ tunnel. Electrochemical performance of (NH_4_)*_x_* WO_3_ electrodes. (k) Areal capacitance as a function of the current densities. (l) Ragone plots of the (NH_4_)*_x_* WO_3_//α‐MnO_2_ A‐HSCs [106] (Copyright 2022, RSC).

Tungsten‐based oxides, particularly WO_3_, have also emerged as a prominent material due to their distinctive physicochemical properties. Its crystal structure and electronic characteristics facilitate unique interactions with NH_4_^+^ ions. A key mechanism is hydrogen bonding between NH_4_^+^ and lattice oxygen atoms of WO_3_. Each NH_4_^+^ can form up to six hydrogen bonds with WO_3_, including three conventional hydrogen bonds and one special trifurcated hydrogen bond. In this trifurcated configuration, a single hydrogen atom from NH_4_^+^ bonds with three oxygen atoms from WO_3_. This interaction leads to the formation of weaker hydrogen bonds between NH_4_^+^ and the lattice oxygen of WO_3_. These weaker bonds help to mitigate the “ion trapping” effect, promote fast NH_4_^+^ diffusion, and reduce structural stress during ion transport, thereby enhancing the electrochemical performance in energy storage and conversion processes (Figure 11a–c) [22, 101, 102].

WO_3_ can form a variety of distinct crystal structures, including monoclinic (*ε*‐WO_3_, *γ*‐WO_3_), orthogonal (*β*‐WO_3_), triclinic (*δ*‐WO_3_), tetragonal (*α*‐WO_3_), and hexagonal (*h*‐WO_3_). Notably, both *h*‐WO_3_ and monoclinic WO_3_ (*m*‐WO_3_) have demonstrated promising performance in AAIBs. Although both structures are composed of WO_6_ octahedra, their spatial arrangements differ significantly. In *m*‐WO_3_, the WO_6_ octahedra are connected through corner sharing, forming a distorted cubic ReO_3_ structure [103, 104]. Chen et al. conducted the first systematic investigation into the formation and phase transition mechanisms of *m*‐WO_3_ nanospheres. They also innovatively revealed the diffusion behavior of NH_4_^+^ during electrochemical processes and the evolution of geometric hydrogen bonding (Figure 11b).

Using a simple hydrothermal method coupled with sintering, they successfully synthesized high‐purity *m*‐WO_3_ nanospheres with excellent crystallinity and dispersion. Electrochemical tests demonstrated that this *m*‐WO_3_ delivers a high specific capacity of 150.6 mA h g^−1^ at a current density of 0.1 A g^−1^ and exhibits excellent rate capability of 48.0 mA h g^−1^ at 5 A g^−1^ (Figure 11d). Furthermore, it showed outstanding cycling stability, maintaining 86.6% of its capacity after 500 cycles. An AAIB constructed with this *m*‐WO_3_ anode and a *γ*‐MnO_2_ cathode achieved a high energy density of 64.9 Wh kg^−1^. Impressively, it retained 95.4% of its capacity after 5000 cycles at 3.0 A g^−1^ (Figure 11e) [102]. In contrast, within *h*‐WO_3_, the WO_6_ octahedra are arranged in hexagonal rings via corner‐sharing oxygen atoms along the (001) plane, forming rigid channels along the *c*‐axis direction [104]. Dong et al. were the first to employ *h*‐WO_3_ as an ammonium ion storage material. Benefiting from its unique structural advantages, *h*‐WO_3_ exhibits significantly enhanced electrochemical performance compared to *m*‐WO_3_. Specifically, water molecules occupying its open channels effectively facilitate NH_4_^+^ transport, and its lattice spacing of 0.31 nm is notably larger than that of *m*‐WO_3_, which is 0.26 nm. At a current density of 1 A g^−1^, *h*‐WO_3_ delivered a high specific capacity of 82.1 mA h g^−1^, which is substantially higher than 14.2 mA h g^−1^ achieved by *m*‐WO_3_. More remarkably, *h*‐WO_3_ demonstrated exceptional long‐term cycling stability, maintaining ∼68% of its capacity after enduring up to 200 000 cycles (Figure 11f,g) [105].

Despite extensive research, their practical performance remains limited by significant volume expansion during charge–discharge cycles and inherently low electrical conductivity. These challenges can lead to structural degradation, poor cycling stability, and sluggish reaction kinetics, hindering efficient ion and electron transport. To address these challenges, researchers have employed NH_4_^+^ pre‐intercalation as an effective strategy to enhance the structural stability and electrochemical performance of these materials. For example, Tang et al. reported a pre‐intercalated NH_4_^+^ stabilized *h*‐WO_3_ anode material for rapid NH_4_^+^ storage. Based on density functional theory (DFT) calculations, they found that the embedded NH_4_^+^ forms hydrogen bonds with adjacent O and H_2_O in WO_3_, and pre‐intercalated NH_4_^+^ significantly enhances the tunnel structures' adsorption capacity for NH_4_^+^. Differential charge density analysis further demonstrated that the interaction between NH_4_^+^ and WO_3_ results in electron enrichment on oxygen atoms. This effect strengthens the hydrogen bonding network and contributes to the structural stabilization of the tunnel‐type WO_3_ framework. Calculations also revealed that NH_4_^+^ pre‐intercalation can reduce its diffusion barrier in the tunnel WO_3_, facilitating rapid and stable storage of NH_4_^+^ (Figure 11h–j). Consequently, the (NH_4_)*_x_* WO_3_ electrode achieves a remarkable areal capacitance of 8.0 F cm^−2^ at 2 mA cm^−2^ which is more than twice that of *h*‐WO_3_ (3.7 F cm^−2^) and WO_3_·0.33H_2_O (3.3 F cm^−2^). Such an outstanding areal capacitance exceeds most previously reported values for hybrid supercapacitor electrodes (Figure 11k). When coupled with a tunnel‐structured *α*‐MnO_2_ cathode, the (NH_4_)*_x_* WO_3_ ammonium‐ion host enables a high‐performance ammonium‐ion hybrid supercapacitor (A‐HSC), achieving an exceptional areal capacitance of 2239.7 mF cm^−2^ and an areal energy density of 1010.1 µW h cm^−2^ (Figure 11l). These findings provide a new prototype for NH_4_^+^ based energy storage and promote a fundamental understanding of the NH_4_^+^ storage mechanism in tunnel‐structured metal oxides [106].

Zhang et al. first synthesized a phosphate ion‐modified tunnel (NH_4_)_0.25_WO_3_ electrode (P‐NWO) using a one‐step hydrothermal method. The NH_4_^+^ pre‐intercalated phosphate‐modified tunnel WO_3_ electrode has dual functions: NH_4_^+^ pre‐intercalation and adsorption of phosphate ions and H_2_O molecules, which enhance the sites for hydrogen bond formation. As a result, P‐NWO delivers a high NH_4_^+^ storage capacity of 148.4 mA h g^−1^ at a current density of 0.2 A g^−1^‐substantially outperforming the unmodified NWO in both capacity and rate capability [107]. This enhanced performance arises from the synergistic effects of NH_4_^+^ pre‐intercalation, phosphate and H_2_O surface adsorption, and optimized hydrogen‐bonding networks that facilitate reversible ion insertion. Oxygen vacancies are intrinsically prevalent in WO_3_ and play a critical role in modulating its electronic structure. These defects act as electron donors, increasing carrier concentration and transforming WO_3_ into an n‐type semiconductor with significantly enhanced electrical conductivity. By tailoring synthesis conditions‐such as annealing temperature and atmosphere (e.g., H_2_/Ar)‐the concentration of oxygen vacancies can be precisely controlled. This tunability enables systematic optimization of electrochemical and catalytic properties. For instance, WO_3−_ *_x_* synthesized at varying temperatures under H_2_/Ar exhibits graded defect densities, allowing for mechanistic studies of vacancy‐dependent photocatalytic activity and charge‐transfer kinetics [108].

### 3.5 Other Related Compounds and Their NH_4_^+^ Storage Mechanism

In addition to the aforementioned V‐, Mn‐, Mo‐, and W‐based layered materials, numerous other related compounds have recently emerged as promising candidates for NH_4_^+^ storage. Their diverse crystallographic architectures and chemical compositions provide rich platforms for developing high‐performance AAIBs and hybrid capacitors.

#### 3.5.1 MXenes: 2D Conductive Hosts for Fast and Stable NH_4_^+^ Storage

**FIGURE 12:**
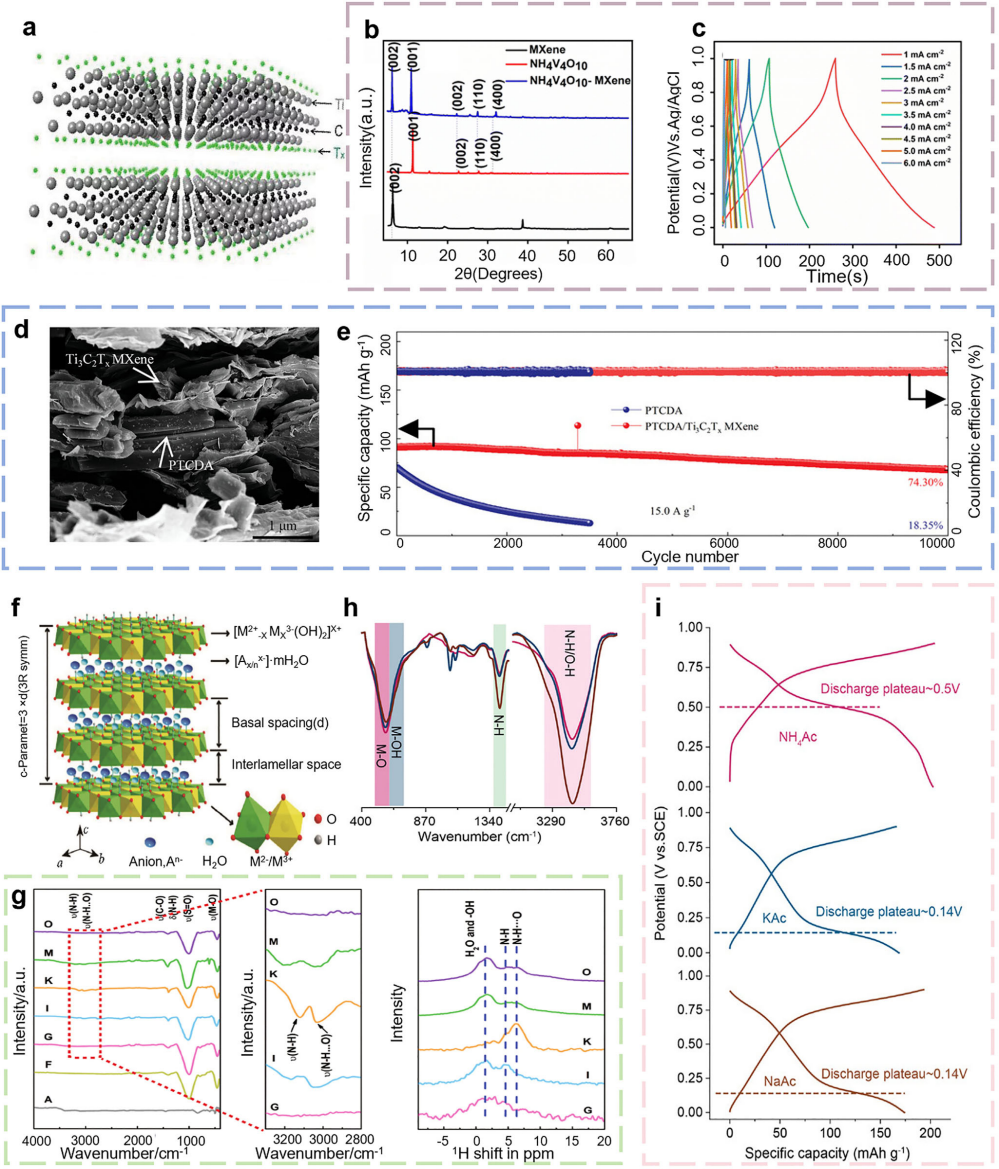
(a) Crystal structure diagrams of MXene [109] (Copyright 2023, Springer). (b) XRD patterns of NH_4_V_4_O_10_, titanium carbide MXene, and NH_4_V_4_O_10_/MXene composite. (c) GCD profiles of NH_4_VO_8_‐MXene electrode at different specific areal current densities [76] (Copyright 2023, Wiley‐VCH). (d) Cross‐sectional SEM image of the PTCDA/MXene freestanding film; (e) Long‐term cycle stability of PTCDA/ MXene at 15.0 A g^−1^ [112] (Copyright 2023, RSC). (f) Crystal structure diagrams of LDHs [113] (Copyright 2023, Wiley‐VCH). (g) Ex situ XRD pattern of Mn_3_Al‐LDHs [36] (Copyright 2023, Elsevier). (h) FTIR spectrum of CoNi‐LDHs. (i) GCD profiles of the CoNi‐LDHs electrode tested in 1 m NH_4_Ac, KAc, and NaAc electrolytes [115] (Copyright 2023, Wiley‐VCH).

Among these, MXenes, 2D transition metal carbides, nitrides, and carbonitrides, stand out due to their exceptional combination of structural tunability and functional versatility. The presence of d‐electrons in the transition metal layers, combined with the efficient electron transport within the metal carbide layers, endows MXene with ultrahigh electrical conductivity comparable to that of metals. Furthermore, its surface is rich in functional groups such as ─F, ─OH, and ═O, which not only enhance electrochemical activity but also improve hydrophilicity and ion adsorption capacity (Figure 12a) [109, 110]. As early as 2013, Gogotsi et al. reported the spontaneous intercalation of various cations, including Na^+^, K^+^, NH_4_^+^, Mg^2+^, and Al^3+^, into the interlayer regions of 2D Ti_3_C_2_T*_x_* MXene. Energy dispersive X‐ray spectroscopy confirmed the presence of NH_4_^+^ within the MXene structure, suggesting that the NH_4_^+^ storage chemistry in MXene holds significant research potential [111]. More recently, researchers have increasingly explored hybrid architectures that integrate MXene with NH_4_^+^‐active host materials to synergistically enhance electrochemical performance. Notably, Krishnan et al. first synthesized a sandwich‐structured composite material composed of ammonium vanadate (NH_4_V_4_O_10_) and Ti_3_C_2_T*_x_* MXene, which was applied as a high‐performance electrode for NH_4_^+^ storage. The NH_4_V_4_O_10_ nanosheets are uniformly anchored or dispersed on the surface or interlayers of the MXene nanosheets, forming a tight heterostructure interface (Figure 12b). This structural design plays a critical role in enhancing the electrochemical activity and stability of the composite. As a result, the NH_4_V_4_O_10_@MXene electrode provides an areal capacitance of 229.0 mF cm^−2^ at a specific current of 1 mA cm^−2^ and retains ∼98% after 5000 charge–discharge cycles (Figure 12c). Furthermore, an A‐HSC assembled with NH_4_V_4_O_8_@MXene as the battery‐type positive electrode and pristine MXene as the capacitive negative electrode delivers a stable energy density of 17.3 Wh kg^−1^ after 10 000 charge–discharge cycles, demonstrating exceptional long‐term cyclability [76]. This robust performance underscores the potential of MXene‐based architectures in durable, high‐efficiency energy storage devices.

Beyond conventional configurations, ammonium‐ion systems have gained increasing attention for next‐generation wearable aqueous micro batteries. The NH_4_^+^ offers several advantageous attributes: low cost, a small hydrated ionic radius (compared to alkali metal ions), fast diffusion kinetics, and a suitable redox potential within the electrochemical stability window of water. These properties enable safe, high‐rate, and flexible energy storage solutions. In this context, Gao et al. developed a self‐supported PTCDA/Ti_3_C_2_T*_x_* film through simple solution mixing and extraction filtration. The resulting binder‐free electrode integrates the reversible NH_4_^+^ storage capability of PTCDA with the metallic conductivity and mechanical robustness of Ti_3_C_2_T*_x_* MXene, forming a tightly coupled, self‐supported architecture (Figure 12d). This hybrid electrode exhibits exceptional cycling stability and outstanding rate performance: it retains 74.3% of its initial capacity after 10 000 charge–discharge cycles and delivers a high specific capacity of 91.67 mAh g^−1^ at a current density of 15.0 A g^−1^, corresponding to a capacity retention of 45.2% when the current density is increased 30‐fold from 0.5 to 15.0 A g^−1^ (Figure 12e) [112].

#### 3.5.2 LDHs: Anion‐Tunable 2D Hosts for Hydrogen‐Bond‐Assisted NH_4_^+^ Storage

Additionally, LDHs have emerged as compelling inorganic hosts for NH_4_^+^ storage, owing to their unique two‐dimensional layered structure and rich multielectron redox chemistry. LDHs consist of positively charged brucite‐like layers composed of edge‐sharing metal hydroxide octahedra, where two or more cations (e.g., Ni^2+^, Co^2+^, Al^3+^, Fe^3+^) are homogeneously distributed, generating a net positive layer charge. Charge neutrality is maintained by intercalated anions (e.g., NO_3_^−^, CO_3_^2−^, Cl^−^) located in the interlayer region. These anions not only balance the charge but also act as structural pillars, effectively expanding the interlayer spacing and creating well‐defined ion diffusion channels. The enlarged interlayer galleries facilitate the reversible intercalation and deintercalation of NH_4_^+^ through a combination of electrostatic interactions, hydrogen bonding (N─H⋯O), and surface‐controlled pseudo capacitance. This structural design significantly enhances ion transport kinetics and electrode stability, rendering LDHs highly suitable for NH_4_^+^ reversible storage (Figure 12f) [113].

Hu et al. Hu et al. first reported the NH_4_^+^ storage characteristics of the MnAl series of LDHs [36]. Utilizing in situ X‐ray photoelectron spectroscopy (XPS) and X‐ray absorption near‐edge structure (XANES) techniques, they accurately captured the reversible valence change of Mn between +3 and +4 states in amorphous Mn_3_Al‐LDH during charge–discharge cycles (Figure 12g). Additionally, through in situ Fourier‐transform infrared spectroscopy (FTIR) and solid‐state nuclear magnetic resonance (NMR), they elucidated the formation and breaking mechanisms of chemical bonds between NH_4_^+^ and metal–oxygen (M–O) sites. The results show that Mn_3_Al‐LDHs electrode achieves a high discharge capacity of 183.8 mA h g^−1^ at 0.1 A g^−1^ and maintains stable cycling at a low working voltage of 0.2 V, underscoring its promise for practical NH_4_^+^‐based energy storage.

Building on this, Wang et al. shifted their focus to amplifying the hydrogen‐bonding effect through defect engineering. They developed a reconstructed CoFe‐LDHs material with engineered oxygen vacancies as a cathode for ammonium‐ion storage. On‐site experimental investigations, together with theoretical calculations, revealed that the presence of structural defects in CoFe‐LDHs significantly reduces the adsorption energy of NH_4_^+^ and induces electron delocalization, thereby enhancing the material's electrical conductivity [114]. As a result, the reconstructed CoFe‐LDHs electrode delivered a reversible capacity of 167.9 mA h g^−1^ at 0.5 A g^−1^, which is 3.3 times higher than that of the pristine CoFe‐LDHs electrode. Inspired by this, Liu et al. introduced hydrogen vacancies and systematically compared the electrochemical storage behavior of NH_4_^+^ with traditional metal ions (Li^+^, K^+^, and Na^+^) [115]. Their findings revealed that, compared to traditional metal ions, using NH_4_^+^ as the charge carrier offers distinct advantages. Not only is the discharge plateau significantly elevated, indicating a higher operating voltage, but the degree of electrochemical polarization is markedly reduced, reflecting faster reaction kinetics and lower internal resistance. This combination is accompanied by a substantial increase in discharge capacity, suggesting enhanced utilization of active sites and improved reversibility of redox processes.

Through an integrated analysis combining experimental characterizations and theoretical simulations, the researchers identified a strong interaction between NH_4_^+^ and electrode materials. In conjunction with the inherently low diffusion barrier of NH_4_^+^, these factors synergistically contribute to the exceptional electrochemical performance observed. Specifically, NH_4_^+^ interacts with the more electronegative oxygen atoms in the electrode structure, forming hydrogen bonds such as Ni─O⋯H─N and Co─O⋯H─N. To further strengthen these hydrogen‐bonding interactions, a substantial number of hydrogen vacancies were intentionally introduced into the material lattice (Figure 12h). This structural modification enhances the electron density of oxygen atoms, thereby increasing their adsorption affinity toward NH_4_^+^ ions. As a result, the CoNi‐LDHs electrode delivers a high discharge capacity of 202.4 mA h g^−1^ at a current density of 0.6 A g^−1^ in NH_4_Ac electrolyte (Figure 12i). Remarkably, the CoNi‐LDHs electrode retains a specific capacity of 176.5 mA h g^−1^ even under high‐current density, corresponding to a capacity retention of ∼72% relative to its low‐current performance, which highlights its excellent rate capability. Furthermore, the assembled AAIBs exhibit a favorable energy density of 123.1 W h kg^−1^ at a power density of 480.0 W kg^−1^, underscoring their promising potential for aqueous high‐performance energy storage applications.

## 4 Summary and Outlook

In summary, AAIBs represent a promising and sustainable energy storage platform, driven by a unique hydrogen‐bond‐mediated ion storage mechanism and the safety of aqueous electrolytes. TMCs stand out as the most widely explored electrode materials for AAIBs owing to their high capacity, favorable operating voltage, competitive energy density, and low cost. A broad range of TMC hosts, including Mn, V, Mo, and W‐based materials, LDHs, and MXenes, exhibit structurally tunable frameworks and high theoretical capacities. Yet these materials commonly face persistent challenges such as structural degradation, sluggish NH_4_^+^ diffusion kinetics, and capacity fade during prolonged cycling. Leveraging the structural and chemical diversity of TMCs, rational material design and advanced electrolytes have achieved notable improvements in cycling performance. Nevertheless, the large ionic radius of NH_4_^+^ continues to pose fundamental constraints on host stability.

Future breakthroughs will depend on engineering robust, adaptive host frameworks capable of accommodating NH_4_^+^ with minimal structural perturbation, paving the way toward practical, high‐performance AAIBs. High‐performance electrode materials remain scarce, while the development of novel full‐cell configurations remains in its early stages. Moreover, the fundamental mechanisms governing hydrogen bond‐mediated charge transfer and dual‐ion cooperative intercalation are not yet fully elucidated. These gaps in knowledge call for the application of advanced in situ and operando characterization techniques, together with theoretical modelling.

**FIGURE 13:**
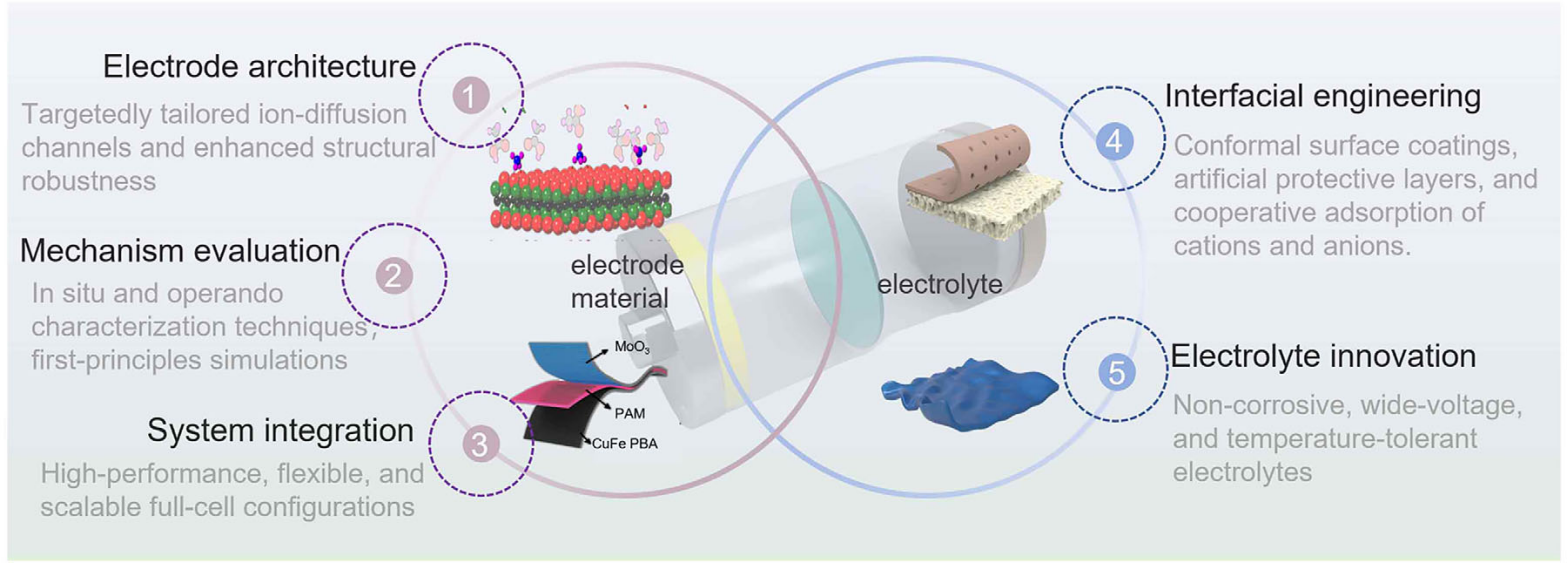
Schematic illustration of promising future research directions on TMCs for AAIBs.

Looking forward, future research should pursue multiple complementary directions to fully unlock their potential (Figure 13). Exploration of novel electrode architectures with tailored ion diffusion channels and enhanced structural robustness is essential for improving rate capability and cycling life. Critically, future development strategies for TMC‐based electrodes must be material‐specific, addressing the distinct limitations inherent to each class of TMCs. Mn‐based oxides are particularly susceptible to Jahn–Teller distortion during repeated redox cycling and can be stabilized through oxygen vacancy engineering or multi‐cation co‐doping strategies, such as Al^3+^/Mg^2+^ incorporation. In contrast, V‐ and Mo‐based compounds suffer from intrinsically poor electronic conductivity and therefore benefit markedly from intimate hybridization with three‐dimensional conductive carbon networks, including graphene foams and carbon nanotube scaffolds. LDHs require effective anion‐pillaring with species such as CO_3_^2−^, NO_3_^−^, and Cl^−^, or heterointerface construction with MXenes, to suppress layer collapse during (de)intercalation. MXenes, despite their high conductivity, demand precise control of surface terminations and effective mitigation of restacking, ideally achieved through intercalant‐assisted delamination, to maximize NH_4_^+^ accessibility and interfacial charge transfer kinetics. At the same time, interfacial engineering at the boundary between electrode and electrolyte will be pivotal in mitigating parasitic side reactions and accelerating charge transfer, particularly under high‐current operation.

A deeper mechanistic understanding is also urgently needed; advanced in situ and operando characterization techniques, combined with first‐principles simulations, will be indispensable for elucidating the microscopic charge storage processes, including hydrogen bond‐mediated interactions and multi‐ion cooperative intercalation. Beyond the materials level, system integration into high‐performance, flexible, and scalable full‐cell configurations will be crucial for translating laboratory findings into real‐world applications. From an industrial perspective, the inherently safe and water‐based chemistry of AAIBs offers significant advantages for large‐scale deployment. Unlike conventional lithium‐ion systems, AAIBs do not require moisture‐sensitive processing or flammable organic electrolytes, thereby eliminating the need for costly dry rooms and simplifying manufacturing logistics. Moreover, many TMCs central to AAIBs are based on earth‐abundant elements such as manganese and molybdenum, which support low material costs and robust supply chains. Recent advances in scalable synthesis methods, including hydrothermal production of LDHs, electrochemical delamination of MXenes, and spray drying of vanadate carbon composites, demonstrate strong compatibility with existing roll‐to‐roll electrode fabrication processes. With coordinated efforts in material design, aqueous slurry formulation, and cell engineering, AAIBs show a realisticpathway toward industrial implementation, leveraging current battery manufacturing infrastructure while delivering enhanced safety, sustainability, and economic viability at scale.

However, there are some critical and practical challenges that must be addressed when translating AAIB technologies into real‐world applications and commercialization. The first issue is the long‐term structural stability of TMC electrodes prepared with high mass loading and thick‐electrode configurations, as repeated NH_4_^+^ insertion and extraction can induce cumulative lattice strain, volume fluctuation, and mechanical degradation. Electronic conductivity is another key bottleneck for TMCs, because the intrinsic electronic conductivity of these materials is generally low, and practical devices therefore rely heavily on conductive carbon additives. During scale‐up, these carbon additives may undergo agglomeration and nonuniform dispersion, leading to heterogeneous current distribution and deteriorated electrochemical performance. Besides, electrolyte compatibility under high current density and prolonged cycling conditions remains another important issue. Addressing these challenges calls for further systematic efforts in materials design, electrode engineering, and device optimization. Finally, the development of advanced electrolytes that are non‐corrosive, wide‐voltage, and temperature‐tolerant electrolytes will be necessary to enhance the practical viability and safety of AAIBs.

In conclusion, although AAIBs are still at an early stage of development, they represent a promising avenue for next‐generation aqueous energy storage systems. With continued interdisciplinary efforts in materials science, electrochemistry, and energy systems engineering, AAIBs are poised to transition from laboratory prototypes to practical energy solutions, contributing meaningfully to the global transition toward clean and sustainable energy technologies.

## Conflicts of Interest

The authors declare no conflicts of interest.

## Data Availbility Statement

The authors have nothing to report.
